# Supplementary material for: Selective CK1α degraders exert antiproliferative activity against a broad range of human cancer cell lines
Source: Nat Commun. 2024 Jan 16;15:482. doi: 10.1038/s41467-024-44698-1 (PMC10791743; doi:10.1038/s41467-024-44698-1)
Supplement: Supplementary file 1 — Supplementary Information [file 41467_2024_44698_MOESM1_ESM.pdf]

SUPPLEMENTARY INFORMATION

Selective CK1α Degraders Exert Antiproliferative Activity Against a Broad Range of Human Cancer Cell Lines

Gisele Nishiguchi[1,9], Lauren G. Mascibroda[2,9], Sarah M. Young[1,9], Elizabeth A. Caine[3], Sherif Abdelhamed[2], Jeffrey J. Kooijman[4], Darcie J. Miller[5], Sourav Das[1], Kevin McGowan[1], Anand Mayasundari[1], Zhe Shi[1], Juan M. Barajas[2], Ryan Hiltenbrand[2], Anup Aggarwal[1], Yunchao Chang[2], Vibhor Mishra[2], Shilpa Narina[6], Melvin Thomas[2], Allister J. Loughran[6], Ravi Kalathur[5], Kaiwen Yu[7], Suiping Zhou[7], Xusheng Wang[7], Anthony A. High[7], Junmin Peng[5,7], Shondra M. Pruett-Miller[6,8], Danette L. Daniels[3], Marjeta Urh[3], Anang A. Shelat[1], Charles G. Mullighan[2], Kristin M. Riching[3], Guido J.R. Zaman[4], Marcus Fischer[1,*], Jeffery M. Klco[2,*] & Zoran Rankovic[1,*]

[1]Department of Chemical Biology and Therapeutics, St. Jude Children's Research Hospital, 262 Danny Thomas Place, Memphis, Tennessee 38105, USA.

[2]Department of Pathology, St. Jude Children's Research Hospital, 262 Danny Thomas Place, Memphis, TN 38105, USA.

[3]Promega Corporation, 5430 East Cheryl Drive, Madison, WI 53711, USA.

[4]Oncolines B.V., Kloosterstraat 9, 5349 AB Oss, The Netherlands

[5]Department of Structural Biology, St. Jude Children's Research Hospital, 262 Danny Thomas Place, Memphis, TN 38105, USA

[6]Center for Advanced Genome Engineering, St. Jude Children's Research Hospital, 262 Danny Thomas Place, Memphis, Memphis, Tennessee 38105, USA.

[7]Center for Proteomics and Metabolomics, St. Jude Children's Research Hospital, 262 Danny Thomas Place, Memphis, TN 38105, USA.

[8]Department of Cell and Molecular Biology, St. Jude Children's Research Hospital, 262 Danny Thomas Place, Memphis, Memphis, Tennessee 38105, USA.

[9]These authors contributed equally to this work.

*Corresponding authors e-mail:

marcus.fischer@stjude.org; jeffery.klco@stjude.org; zoran.rankovic@stjude.org.

Table of Contents

1. Supplementary Figures
2. Supplementary Tables
3. Supplementary Methods
4. References
5. Uncropped blots

Supplementary Figures

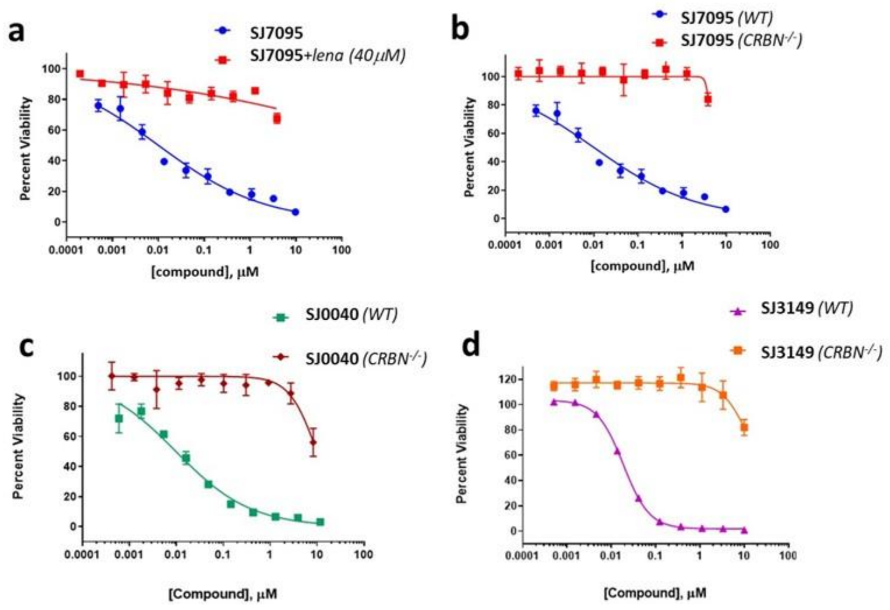

Supplementary Fig. 1| CRBN-dependency of CK1α degrader cytotoxicity in MOLM-13 cell line. a. Effect of SJ7095 on MOLM-13 viability in the absence and presence of lenalidomide (40 µM). **b.** Effect of SJ7095 on viability of wild type and $CRBN^{-/-}$ MOLM-13 cells. **c.** Effect of SJ0040 on viability of wild type and $CRBN^{-/-}$ MOLM-13 cells. **d.** Effect of SJ3149 on viability of wild type and $CRBN^{-/-}$ MOLM-13 cells. Data represents 3 independent experiments and error bars indicate SEM.

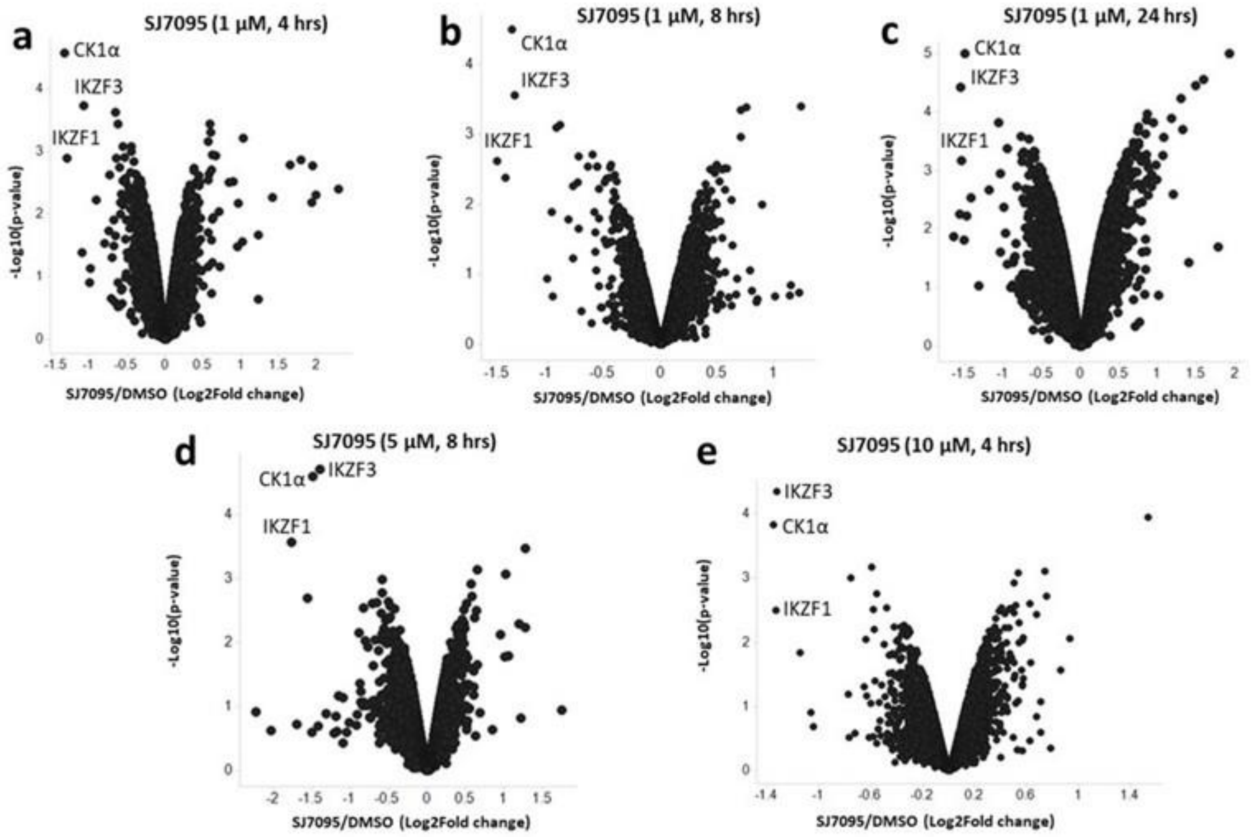

Supplementary Fig. 2| TMT proteomics in MOLM-13 after incubation with SJ7095 at different concentrations and timepoints. a. 1 µM, 4 h. **b.** 1 µM, 8 h. **c.** 1 µM, 24 h. **d.** 5 µM, 8 h. **e.** 10 µM, 4 h. Each treatment was run in duplicate. Statistical analysis: two-tailed and unpaired t-test.

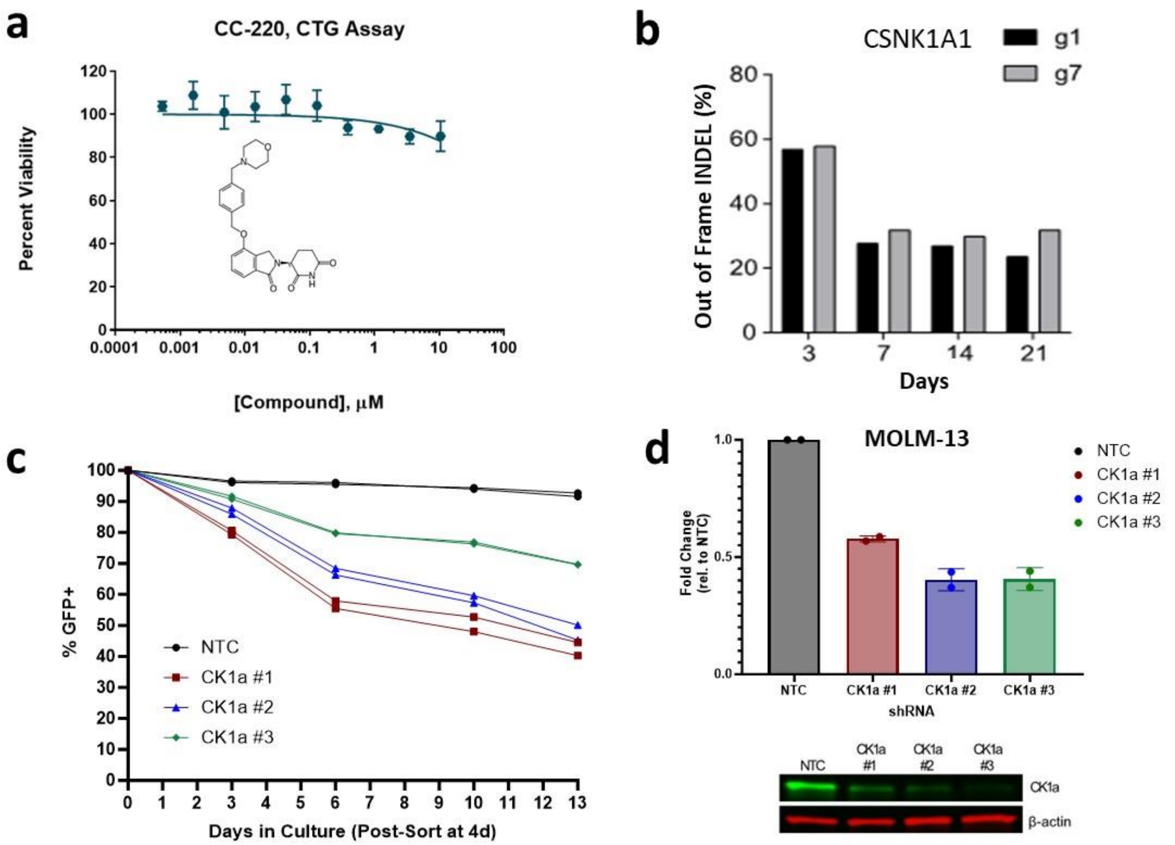

Supplementary Fig. 3| MOLM-13 cells are highly dependent on CK1α expression. a. MOLM-13 cells viability measured in the CTG assay after 72 h incubation with rising concentrations of iberdomide (CC-220). Data represents 3 independent experiments and error bars indicate SEM. **b.** CRISPR genomic editing of MOLM-13 cells using RNA guides against CK1α and determining the relative change of out-of-frame INDEL (insertions or deletions) frequency at different time points (n=1). **c.** The percentage of GFP positive cells measured over time following shRNA-targeted silencing of CK1α. Data represents the average of 2 independent determinations. Error bars indicate standard deviation. **d.** Representative immunoblot of CK1α expression after shRNA treatment and corresponding relative expression of 2 independent determinations. Error bars indicate standard deviation.

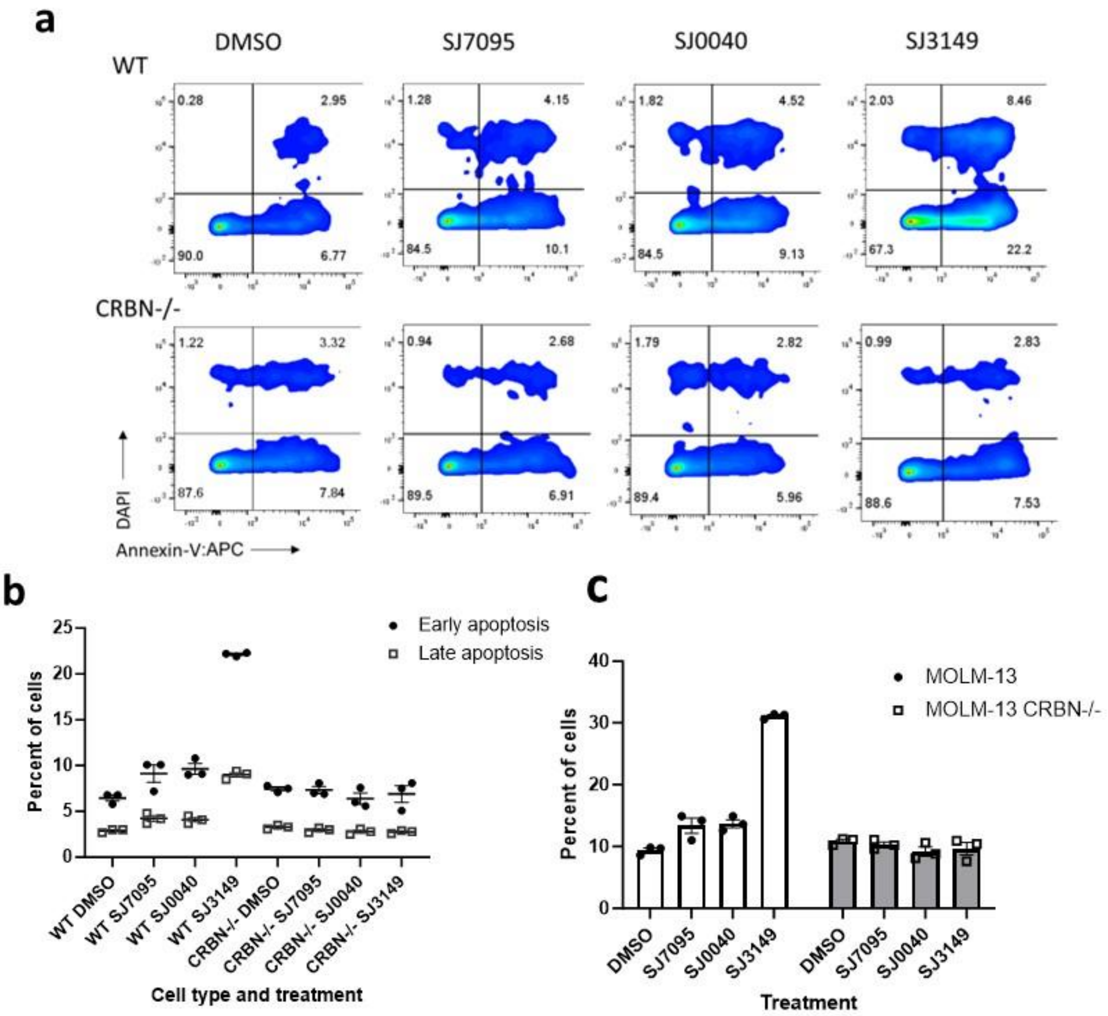

Supplementary Fig. 4| Annexin V staining data in wild type MOLM-13 and CRBN$^{-/-}$ MOLM-13 cells after 24 hours incubation. a. Representative images of triplicate flow cytometry results for annexin V cell surface expression. The x-axis is annexin V (APC) signal and the y-axis is DAPI signal. For each plot, the lower right quadrant is early apoptosis, and the upper right quadrant is late apoptosis. Gating strategy is explained in Supplementary Figure 13. **b.** Stacked scatterplot showing the percentage of cells in each apoptotic stage. Bars indicate means and SEM values. **c.** Bar graph showing the mean total percentage of apoptotic cells in each condition. Error bars indicate standard error of the mean.

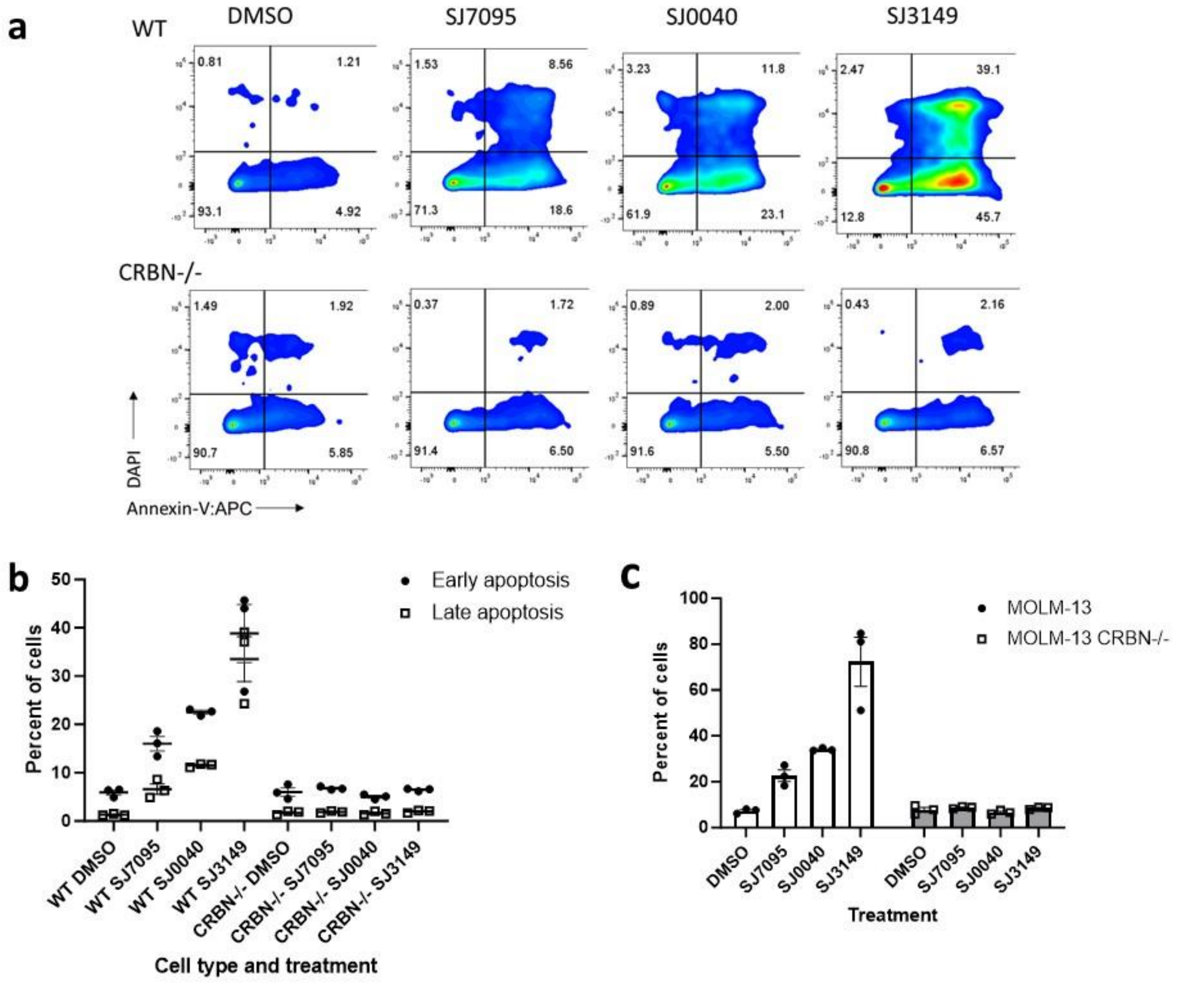

Supplementary Fig. 5| Annexin V staining data in wild type MOLM-13 and CRBN$^{-/-}$ MOLM-13 cells after 72 hours incubation. a. Representative images of triplicate flow cytometry results for annexin V cell surface expression. The x-axis is annexin V signal and the y-axis is DAPI signal. For each plot, the lower right quadrant is early apoptosis, and the upper right quadrant is late apoptosis. Gating strategy is explained in Supplementary Figure 13. **b.** Stacked scatterplot showing the percentage of cells in each apoptotic stage. Bars indicate means and SEM values. **c.** Bar graph showing the mean total percentage of apoptotic cells in each condition. Error bars indicate standard error of the mean.

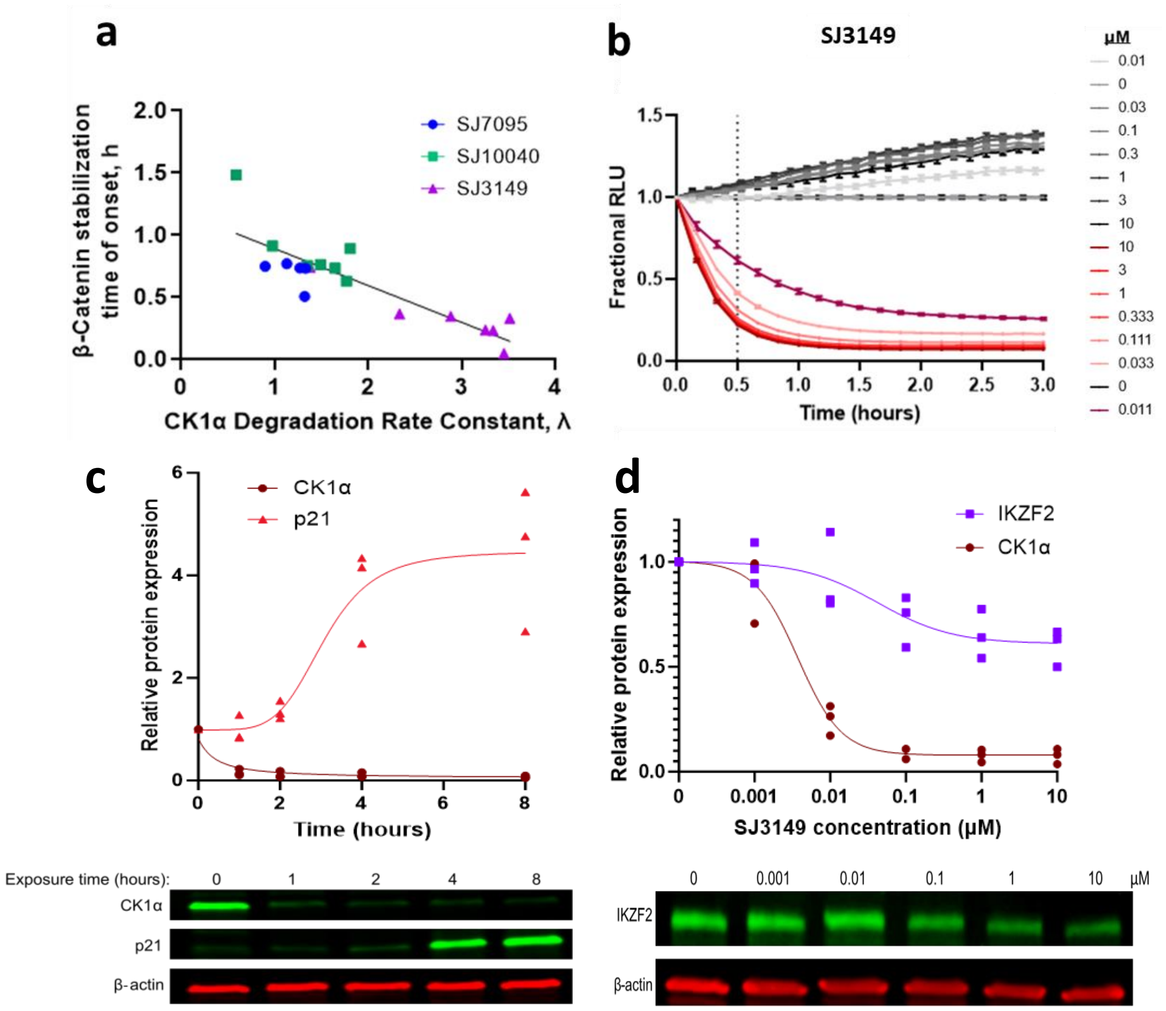

Supplementary Fig. 6| Downstream and off-target profiling of compounds. a. β-Catenin stabilization time of onset compared to CK1α degradation rate constant for SJ7095, SJ0040 and SJ3149. **b.** CK1α (shades of red) and β–catenin (shades of grey/black) protein changes in HiBiT (HEK293) cells as function of time and concentration. Data represents 4 technical replicates. **c.** MOLM-13 cells treated with 1 μM SJ3149 from 0-8 hours lysed and analyzed by western blot. Representative blot showing expression of CK1α, p21, and β-actin over time. Relative expression of CK1α and p21 is determined from 3 independent experiments. (Curve calculated by least squares nonlinear fit, R2=0.9148 for CK1α and 0.8506 for p21.) **d.** Relative IKZF2 and CK1α protein levels in MOLM-13 cells after 4 hours of treatment with SJ3149 at increasing concentrations. CK1α data is from experiment shown in Fig. 2b. Representative western blot showing expression of IKZF2 and β-actin. Data shown is from three independent determinations (curve calculated by least squares nonlinear fit, R2=0.7346 for IKZF2, 0.9760 for CK1α.)

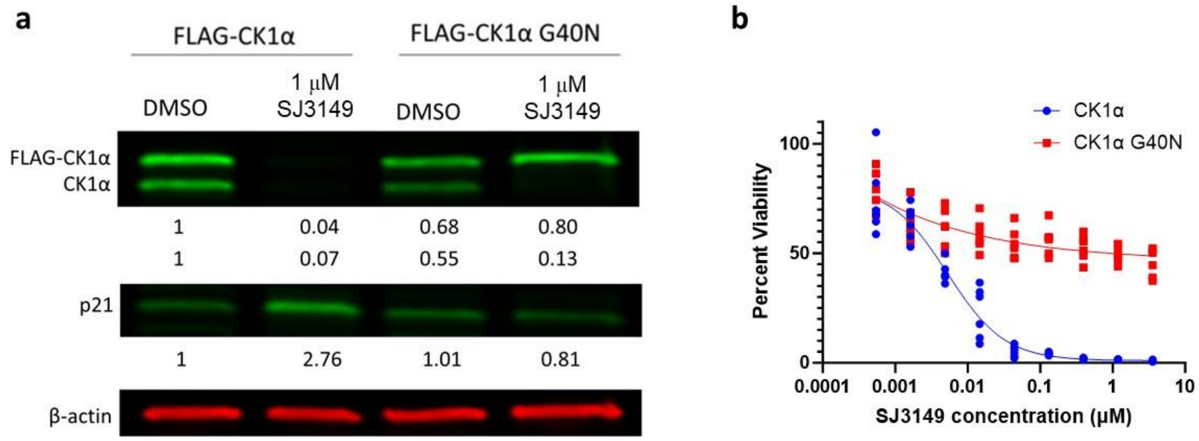

Supplementary Fig. 7| Impact of mutant CK1α on SJ3149 phenotype in MOLM-13 cells. a. Immunoblot of transduced MOLM-13 cells after 4-hour treatment with DMSO or 1 μM SJ3149. Cells expressing FLAG-CK1α are shown in the left 2 lanes, and cells expressing the FLAG-CK1α G40N mutant are in the right 2 lanes. Lysates were probed for CK1α, p21, and β-actin. (n=1) **b.** Cell viability as measured by CellTitre Glo luminescence assay in FLAG-CK1α overexpression MOLM-13 cells. Cells were treated with increasing concentrations of SJ3149 and incubated for 3 days before viability measurements. (n=6, from 2 independent experiments with 3 replicates each; curve calculated by least squares nonlinear fit, R^2=0.9381 for CK1α, 0.6167 for CK1α G40N.)

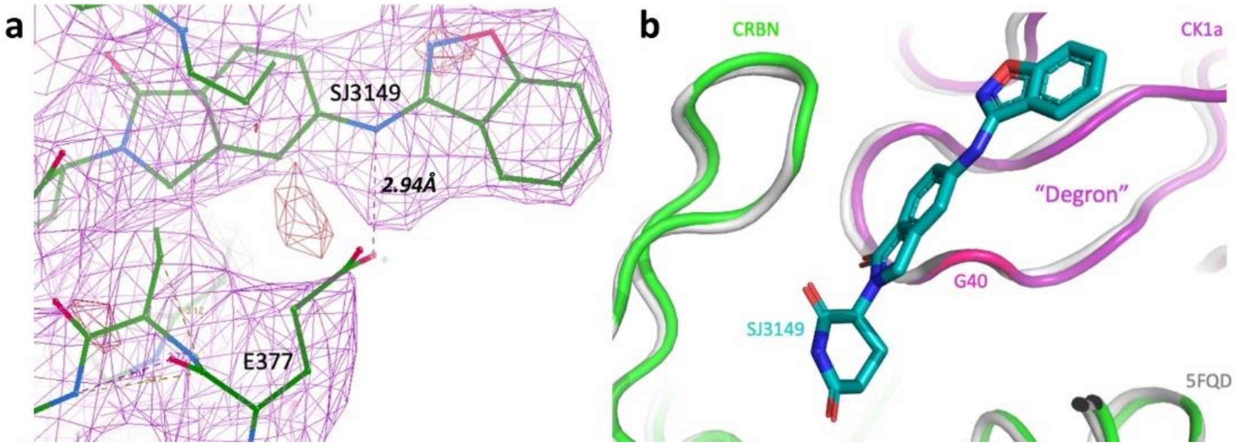

Supplementary Fig. 8| SJ3149 bound at the interface of CRBN and CK1α: a. Electron density evidence for the direct interaction between E377 and SJ3149 (rendered at 0.82 sigma). **b.** Ribbon diagram of SJ3149 (cyan sticks) at the interface of CRBN (green) and CK1α (purple) overlaid onto 5FQD (grey). SJ3149 contacts the CK1α "degron" loop (purple), including G40 (pink) that is essential for recruitment of CK1α to CRBN.

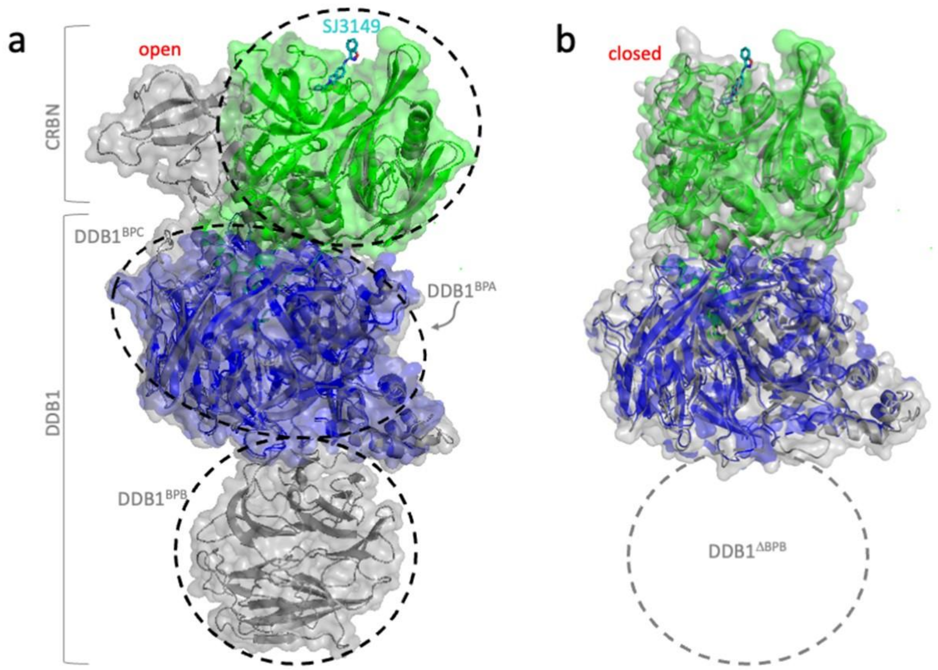

Supplementary Fig. 9| Comparison of the “open” (PDB: 8CVP) and “closed” (PDB: 8D81) states of CRBN (4) shows that CRBN adopts a closed conformation in the CK1α+CRBN+DDB1+ligand quaternary complex. **a.** Overlay of open CRBN (8CVP; grey) depicts CRBN in the “open” state with poor overlap of CRBN (green) from our model illustrating that CRBN from our model exists in the “closed” conformation. Full-length DDB1 of 8CVP (grey) overlaid with DDB1$^{\Delta BPB}$ (blue) from our structure remains largely unchanged. SJ3149 (cyan sticks) shows binding to the thalidomide binding domain of CRBN (green) and interaction with the β-hairpin “sensor loop”. The benzisoxazol of SJ3149 is solvent exposed. Black dashes indicate domain boundaries; CK1α was excluded for clarity. **b.** The structural overlap of the domains of “closed” CRBN (8D81; grey) with CRBN$^{\Delta 1\text{-}40}$ (green) and DDB1$^{\Delta BPB}$ (blue) shows that CRBN is in the “closed” state.

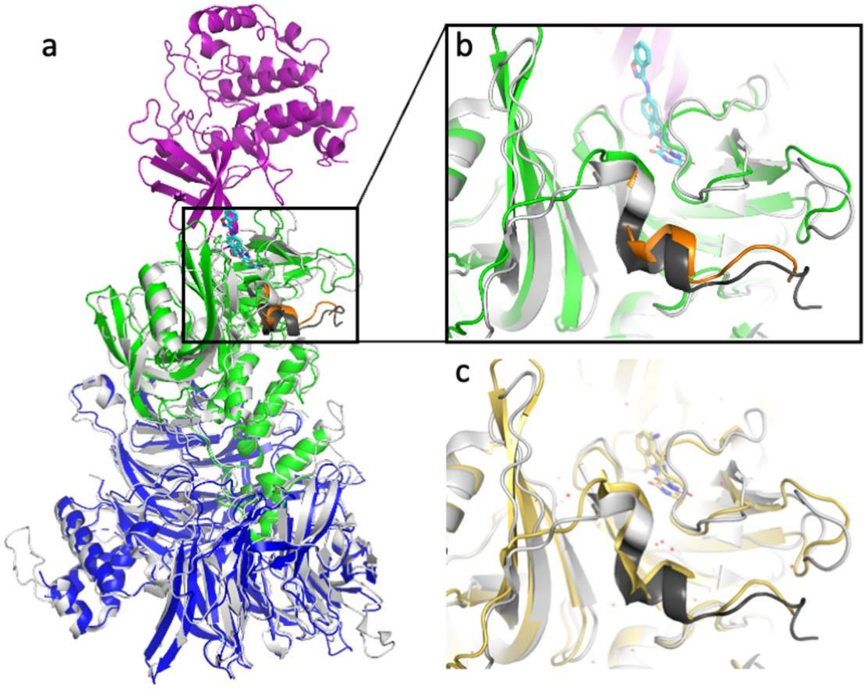

Supplementary Fig. 10| The N-terminal "belt" (orange) of the Lon domain wraps around the thalidomide binding domain (TBD) to stabilize the closed CRBN conformation. **a.** Closed CRBN-DDB1 complex (8D81; grey, with safety belt shown in dark grey) bound to Pomalidomide (grey sticks) superimposed onto our quaternary structure with domain colors as above, SJ3149 shown as cyan sticks, and belt region shown in orange. **b-c** Zoom of superposition for **b** our SJ3149-bound structure and **c** Lenalidomide-bound 5FQD (yellow), both showing a closed belt.

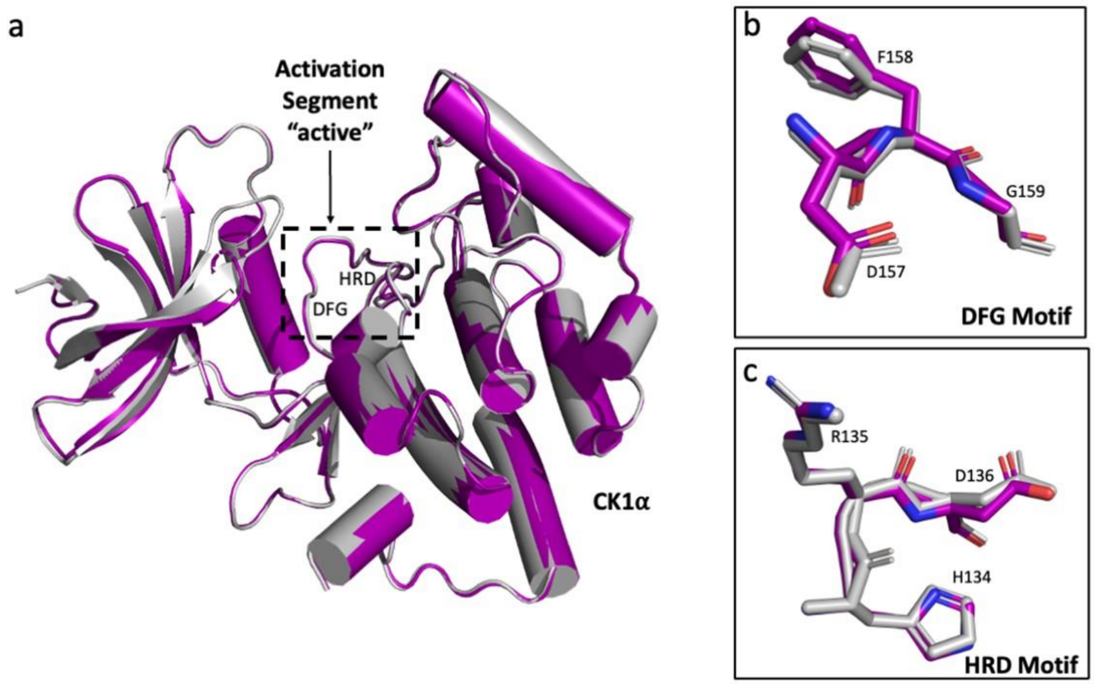

Supplementary Fig. 11| CK1α in the quaternary complex is in the active state. **a.** Overlay of our CK1α (purple) with active CK1α from 5FQD (grey); other components of the quaternary complex are excluded for clarity. The disordered activation segment depicting the active conformation of CK1α is highlighted within the dashed box. CK1α’s DFG motif (**b**) and HRD motif (**c**) are in the “active” conformation.

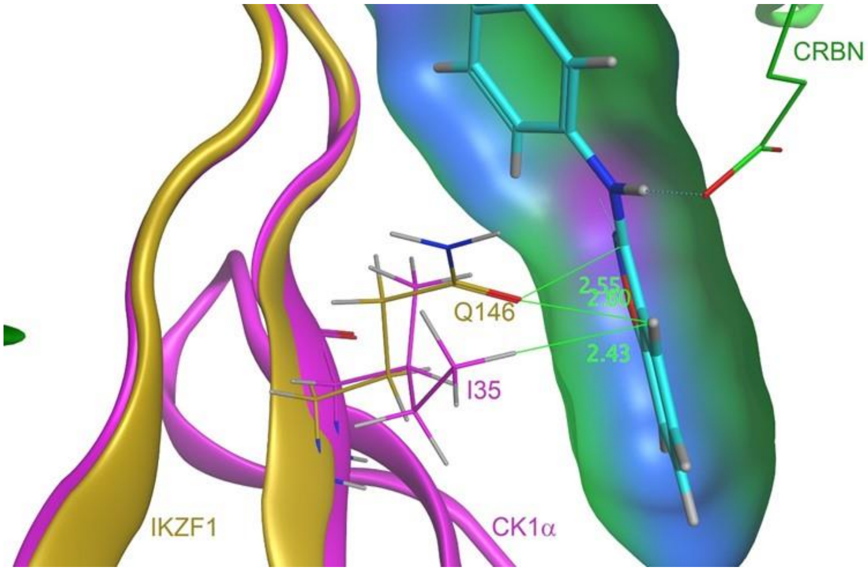

Supplementary Fig. 12| IKZF1 from the IKZF1-CRBN-DDB1 (PDB: 6H0F7) complex is aligned onto the CK1α protein in the SJ3149-CK1α-CRBN-DDB1 complex. SJ3149 is depicted by its property-mapped Gauss-Connolly molecular surface, where green represents hydrophobic patches and blue and purple represent polar patches [Molecular Operating Environment (MOE) 2022.2]. Distances (Å) are shown in green. I35 of CK1α is positioned next to the hydrophobic patch created by the six-membered aromatic carbon ring of SJ3149. In IKZF1, Q146, which is a polar residue, is positioned next to the hydrophobic patch of the ligand. This incompatibility could be responsible for the inactivity of Series SJ3149 in IKZF1. IKZF1 is shown in gold, CRBN in green and CK1α in purple.

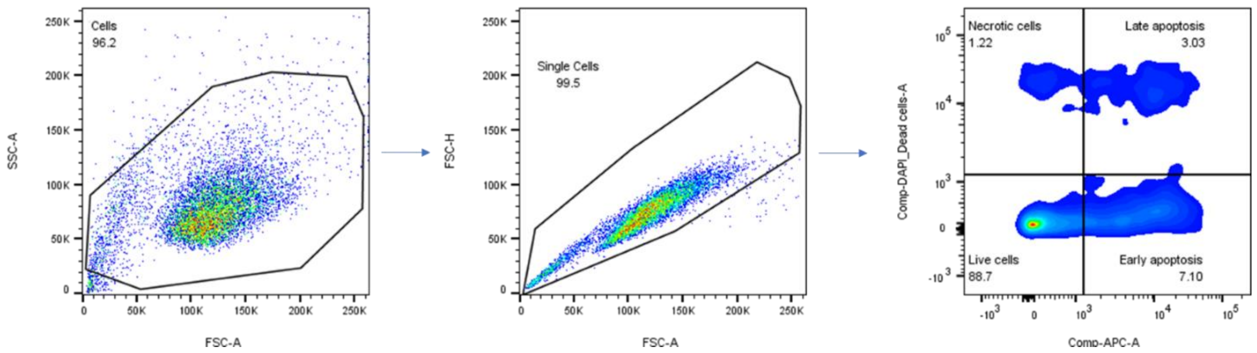

Supplementary Fig. 13: Gating for Annexin V flow cytometry analysis. Total cells were gated to remove cell debris, followed by single cell gating on the FSC-A/FSC-H diagonal. Within the single cell population, quadrants were divided at the 10^3 point on both axes, DAPI and Annexin V: APC. Live cells are negative for both fluorescent markers, DAPI positive/APC negative cells are necrotic, APC positive/DAPI negative cells are still alive but in the early stages of apoptosis, and double positive cells are dead via apoptosis. Percentages of early and late apoptosis were plotted in supplementary figures 4 and 5.

Supplementary Fig. 14: 1H NMR, 13C NMR, LCMS and HRMS for all synthesized compounds (SJ7095, SJ0040 and SJ3149).

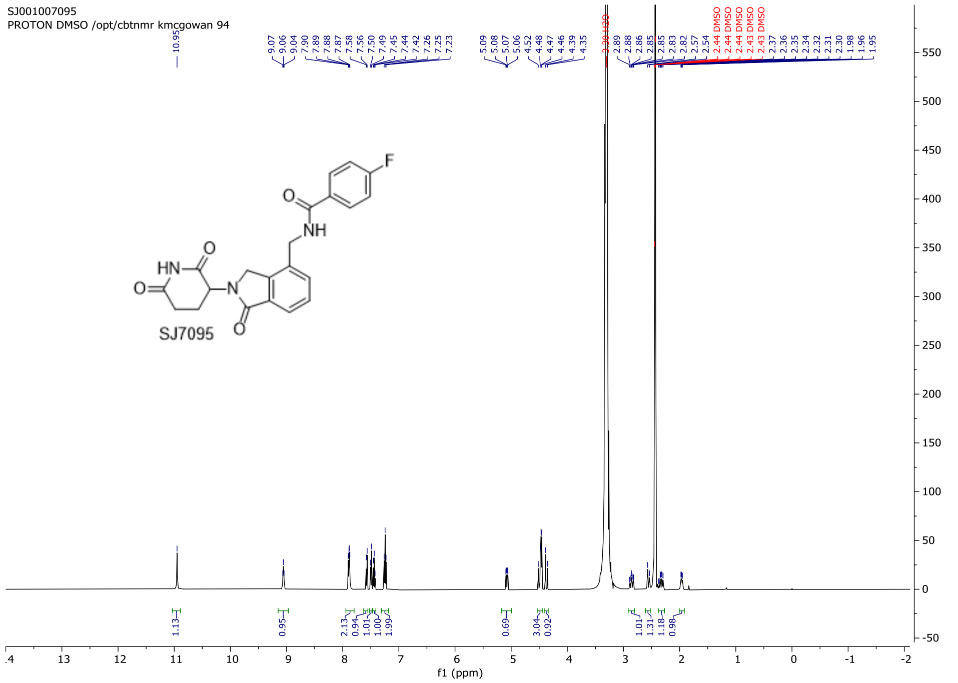

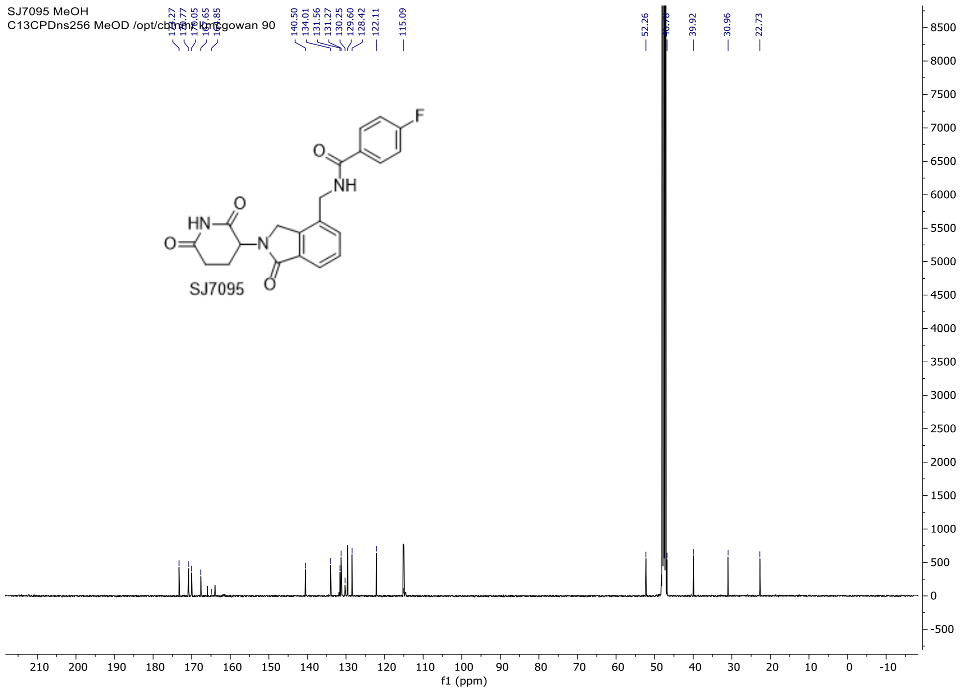

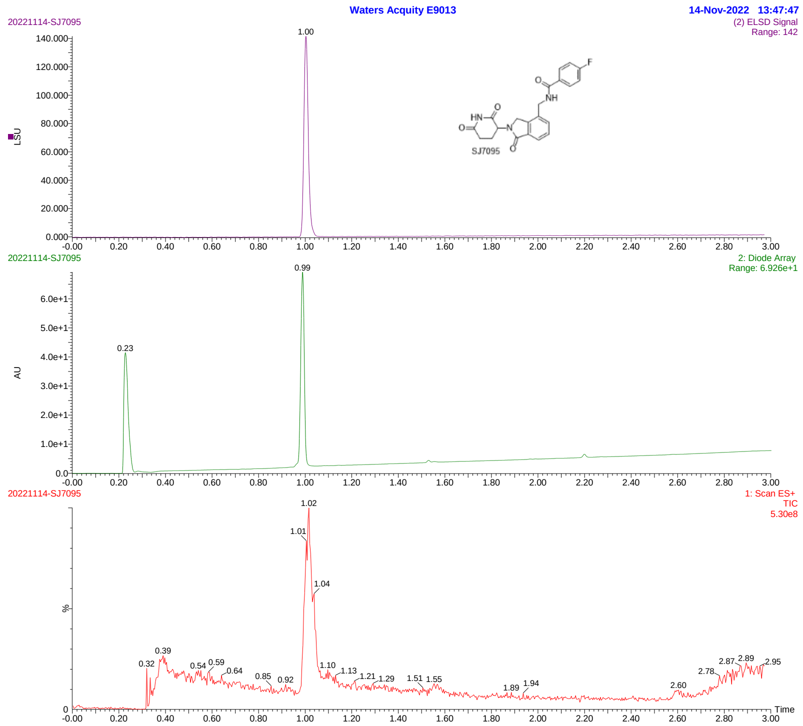
Waters Acquity E9013
14-Nov-2022 13:47:47
20221114-SJ7095
(2) ELSD Signal
Range: 142
LSU
1.00
SJ7095
20221114-SJ7095
2: Diode Array
Range: 6.926e+1
AU
0.99
0.23
20221114-SJ7095
1: Scan ES+
TIC
5.30e8
%
1.02
1.01
1.04
Time

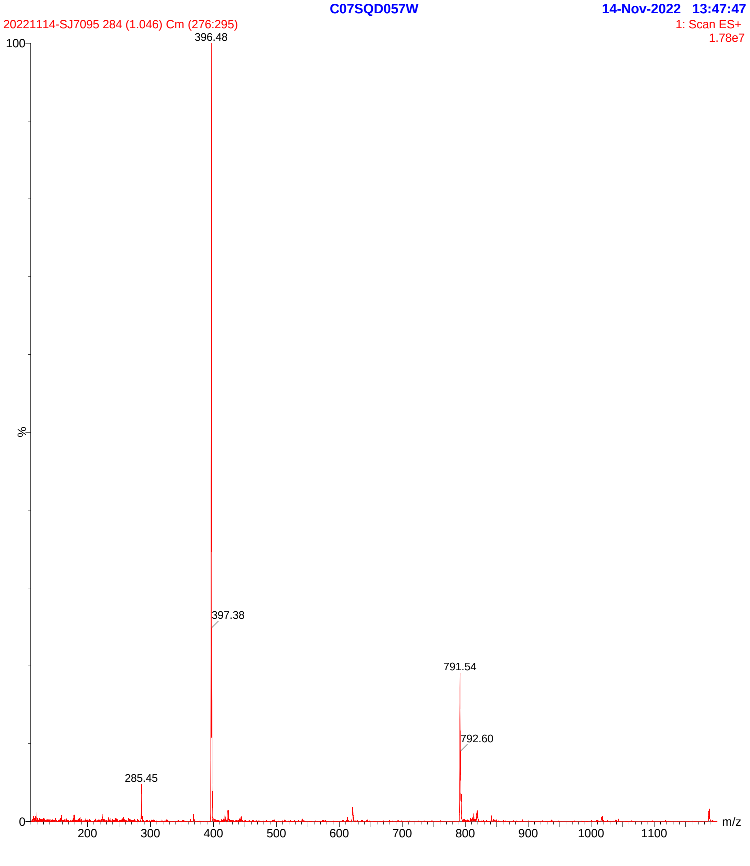
C07SQD057W
14-Nov-2022 13:47:47
20221114-SJ7095 284 (1.046) Cm (276:295)
1: Scan ES+
1.78e7
396.48
397.38
791.54
792.60
285.45
%
m/z

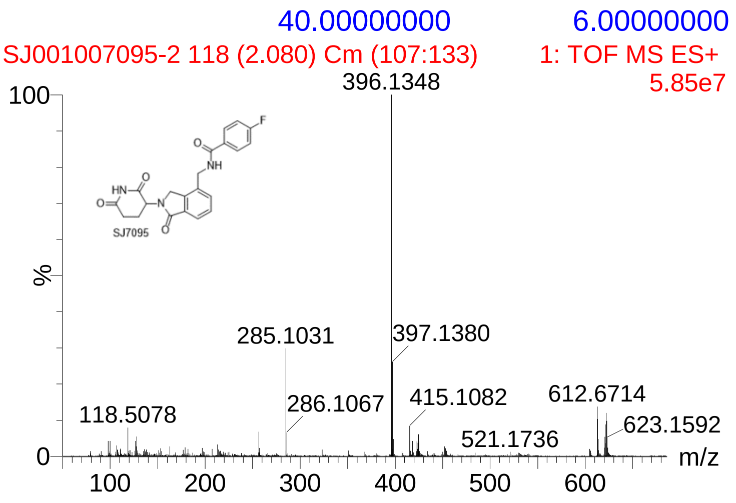
40.00000000
6.00000000
SJ001007095-2 118 (2.080) Cm (107:133)
1: TOF MS ES+
5.85e7
396.1348
397.1380
285.1031
286.1067
415.1082
612.6714
623.1592
521.1736
118.5078
SJ7095
%
m/z

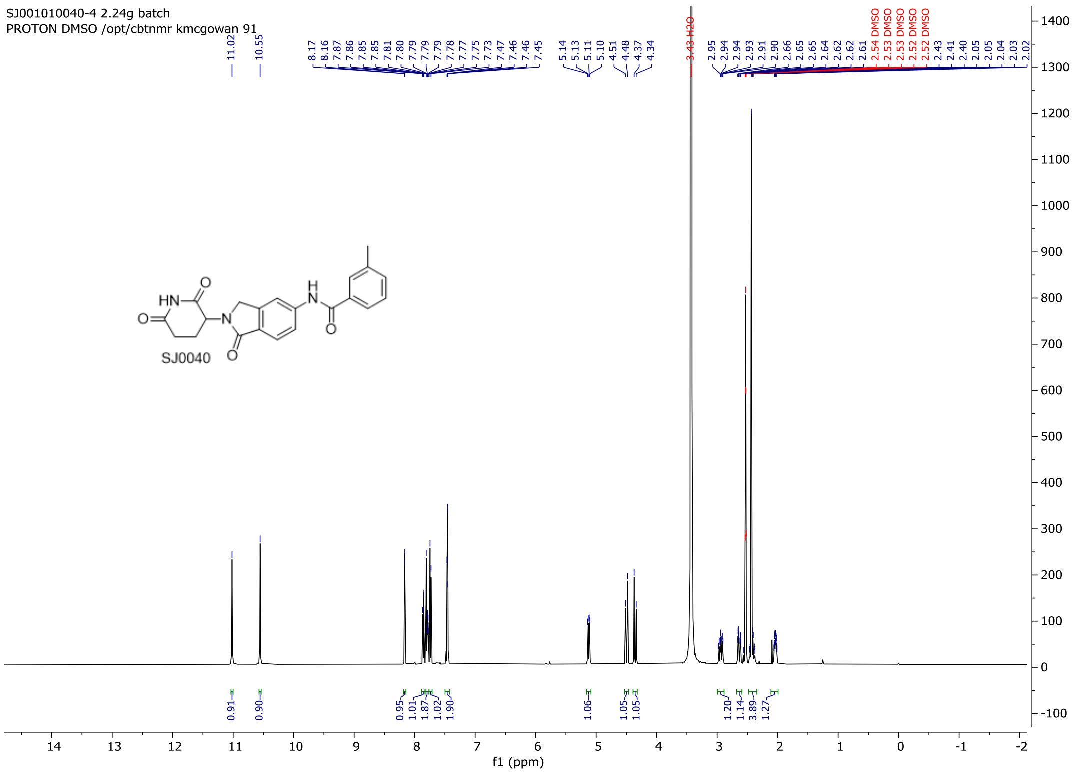
SJ001010040-4 2.24g batch
PROTON DMSO /opt/cbtnmr kmcgowan 91
11.02
10.55
SJ0040
f1 (ppm)

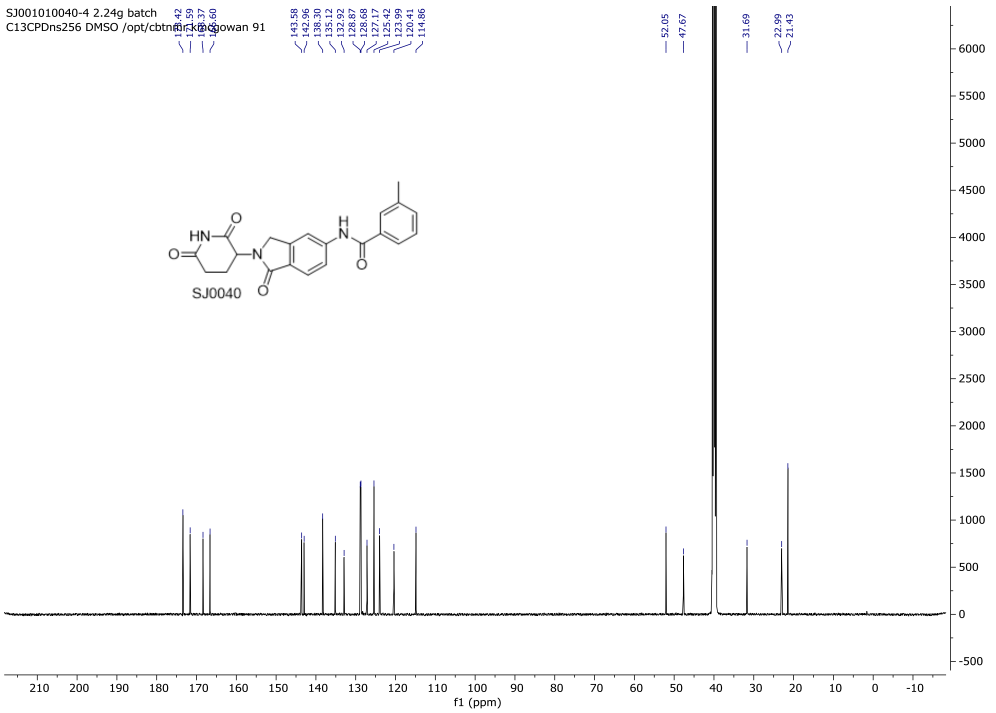
SJ001010040-4 2.24g batch
C13CPDns256 DMSO /opt/cbtnmr kmcgowan 91
173.42
171.59
168.37
166.60
143.58
142.96
138.30
135.12
132.92
128.87
128.68
127.17
125.42
123.99
120.41
114.86
52.05
47.67
31.69
22.99
21.43
SJ0040
f1 (ppm)

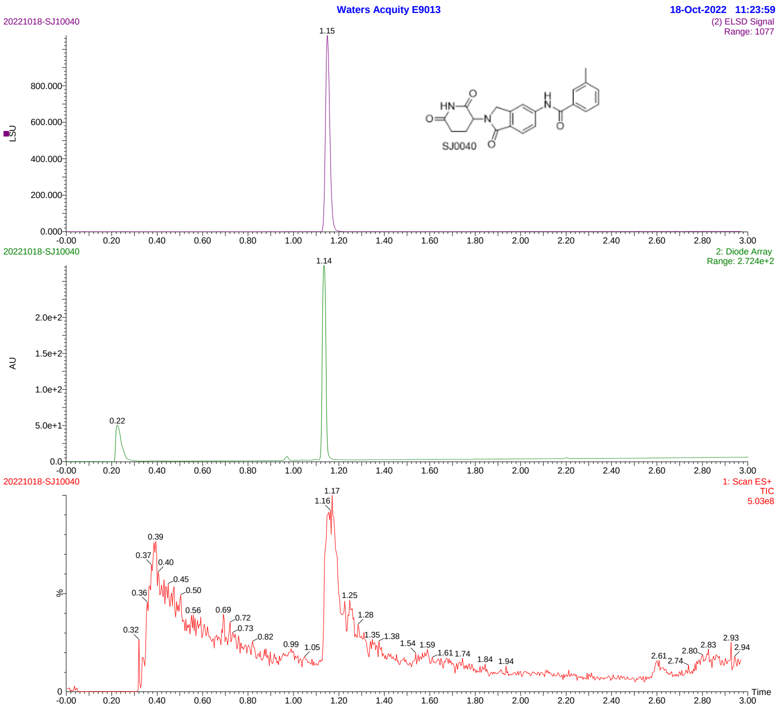
Waters Acquity E9013
18-Oct-2022 11:23:59
20221018-SJ10040
(2) ELSD Signal
Range: 1077
LSU
SJ0040
HN
N
O
1.15
20221018-SJ10040
2: Diode Array
Range: 2.724e+2
AU
0.22
1.14
20221018-SJ10040
1: Scan ES+
TIC
5.03e8
%
Time
0.32
0.36
0.37
0.39
0.40
0.45
0.50
0.56
0.69
0.72
0.73
0.82
0.99
1.05
1.16
1.17
1.25
1.28
1.35
1.38
1.54
1.59
1.61
1.74
1.84
1.94
2.61
2.74
2.80
2.83
2.93
2.94

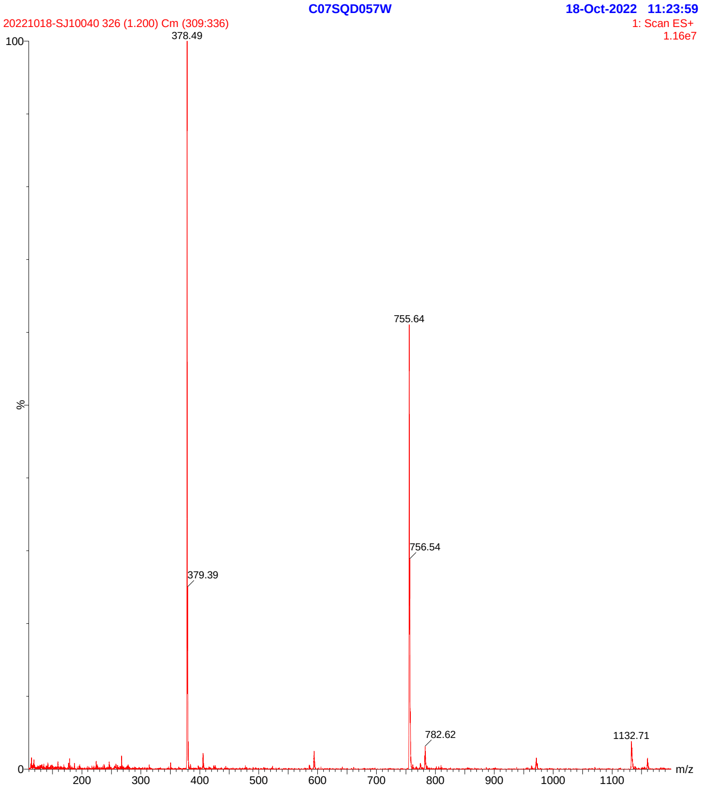
C07SQD057W
18-Oct-2022 11:23:59
20221018-SJ10040 326 (1.200) Cm (309:336)
1: Scan ES+
1.16e7
%
m/z
378.49
379.39
755.64
756.54
782.62
1132.71

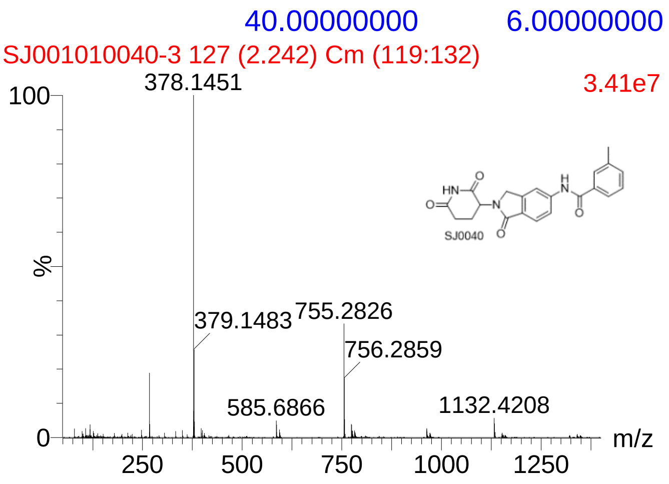
40.00000000
6.00000000
SJ001010040-3 127 (2.242) Cm (119:132)
3.41e7
378.1451
379.1483
755.2826
756.2859
585.6866
1132.4208
%
m/z
SJ0040

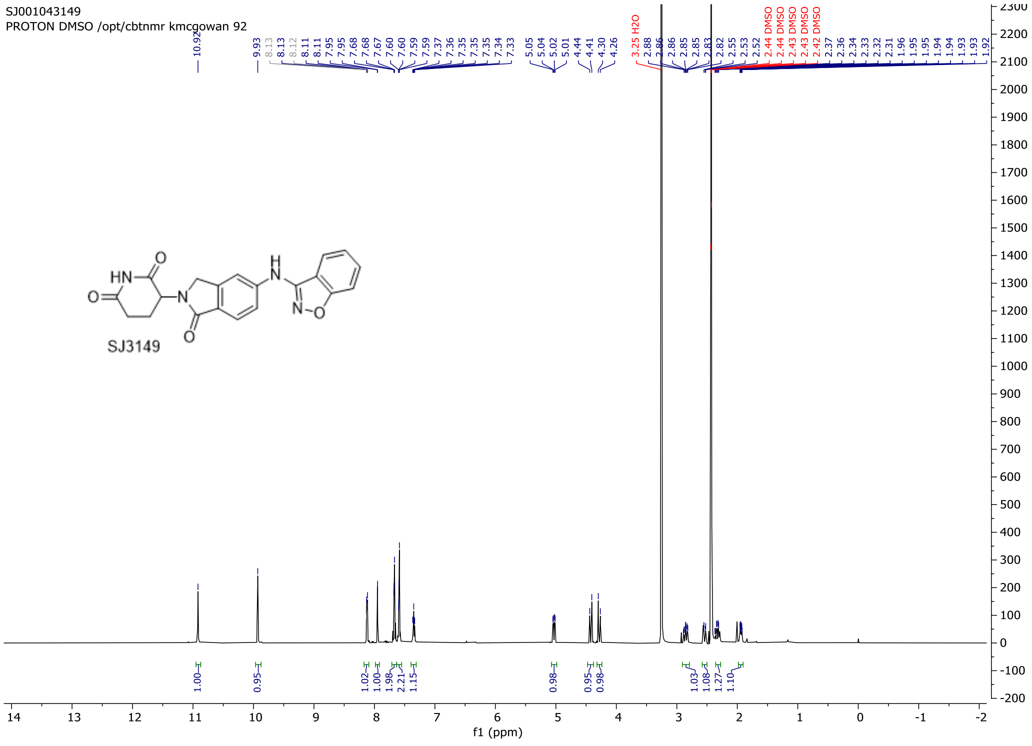
SJ001043149
PROTON DMSO /opt/cbtnmr kmcgowan 92
10.92
9.93
8.13 8.13 8.12 8.11 8.11 7.95 7.95 7.68 7.67 7.60 7.60 7.59 7.59 7.37 7.36 7.35 7.35 7.34 7.33
5.05 5.04 5.02 5.01 4.44 4.41 4.30 4.26
3.25 H2O
2.88 2.86 2.86 2.85 2.85 2.83 2.82 2.55 2.53 2.52
2.44 DMSO 2.44 DMSO 2.43 DMSO 2.43 DMSO 2.42 DMSO
2.37 2.36 2.34 2.33 2.32 2.31 1.96 1.95 1.95 1.94 1.94 1.93 1.93 1.92
1.00 0.95 1.02 1.00 1.98 2.21 1.15 0.98 0.95 0.98 1.03 1.08 1.27 1.10
SJ3149
f1 (ppm)

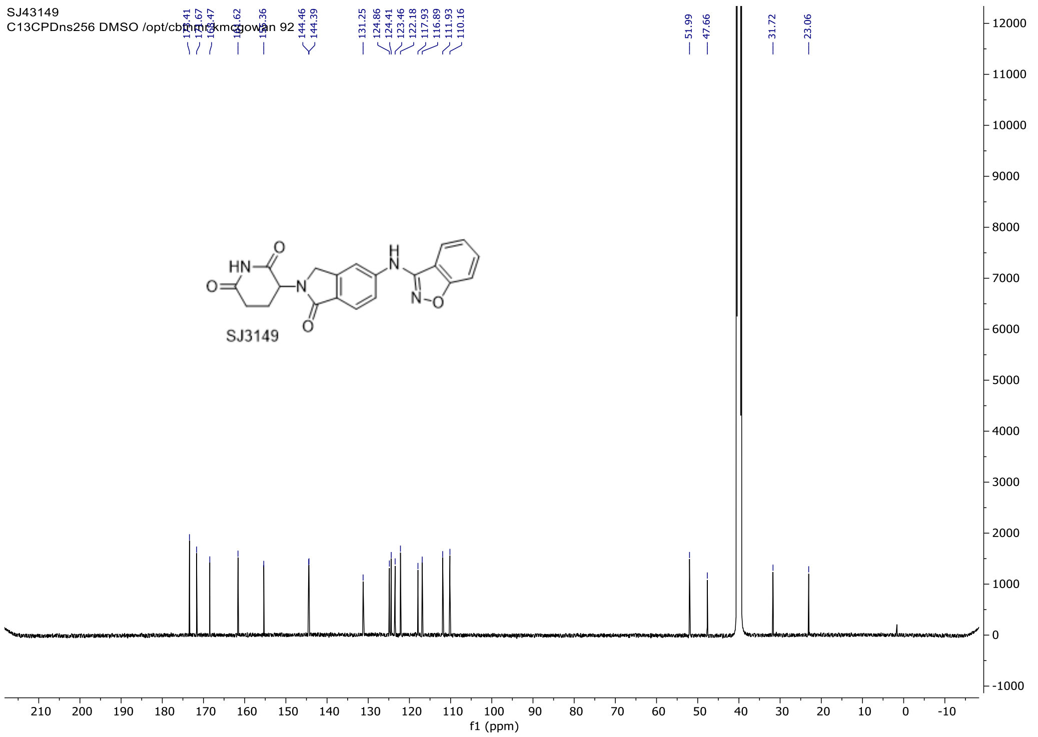
SJ43149
C13CPDns256 DMSO /opt/cbtnmr/kmcgowan 92
161.62
144.46
144.39
131.25
124.86
124.41
123.46
122.18
117.93
116.89
111.93
110.16
51.99
47.66
31.72
23.06
HN
O
N
H
N
N
O
SJ3149
f1 (ppm)

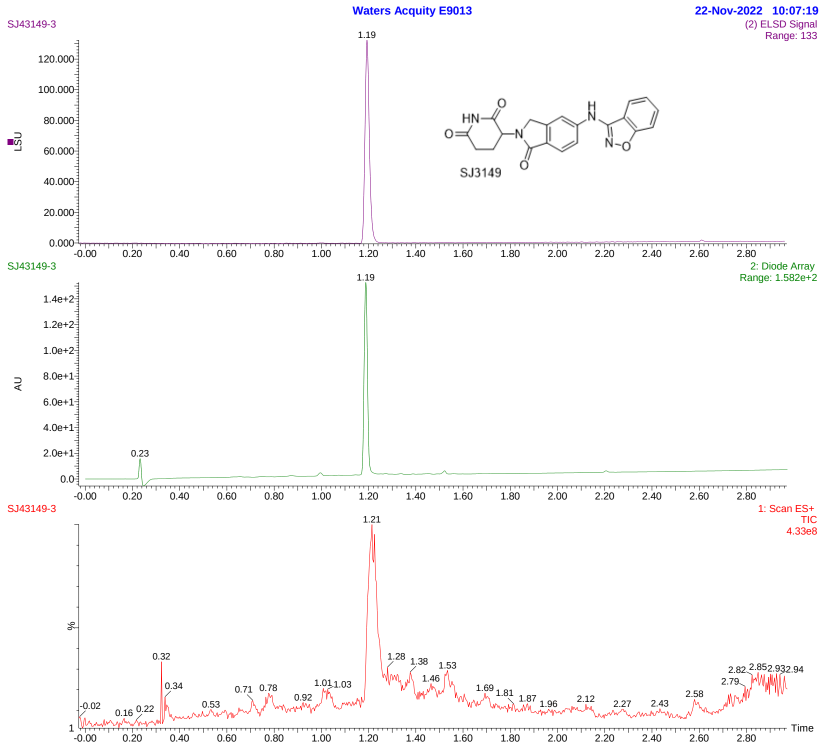
Waters Acquity E9013
22-Nov-2022 10:07:19
SJ43149-3
(2) ELSD Signal
Range: 133
LSU
SJ3149
SJ43149-3
2: Diode Array
Range: 1.582e+2
AU
SJ43149-3
1: Scan ES+
TIC
4.33e8
%
Time

C07SQD057W 22-Nov-2022 10:07:19

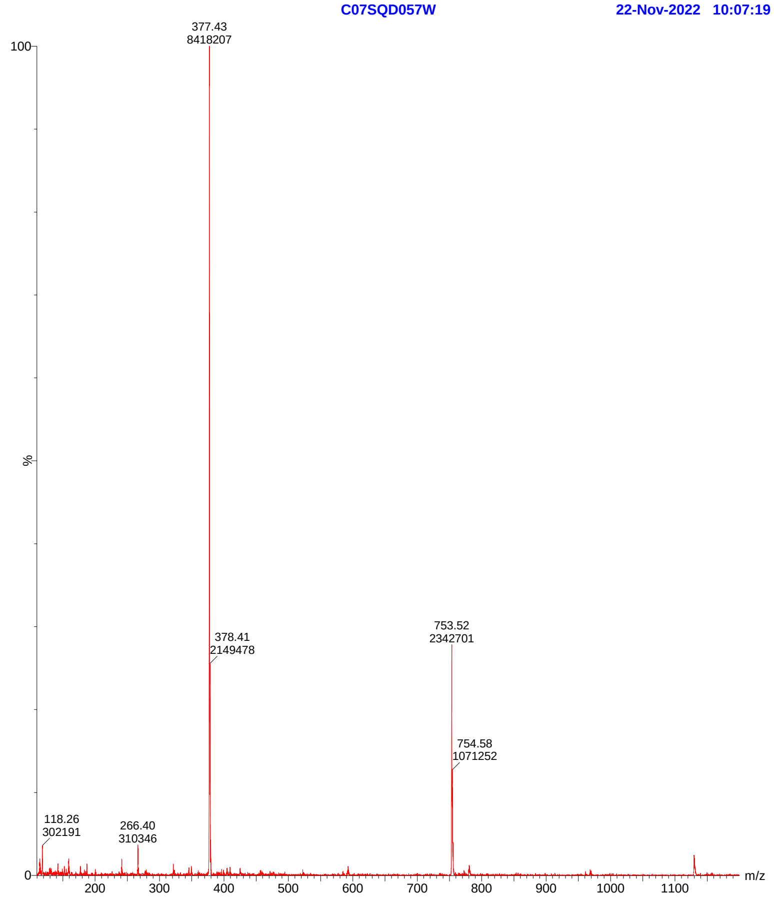

40.00000000 6.00000000

SJ001043149-1_20230222 229 (4.016) Cm (223:236)

6.43e7

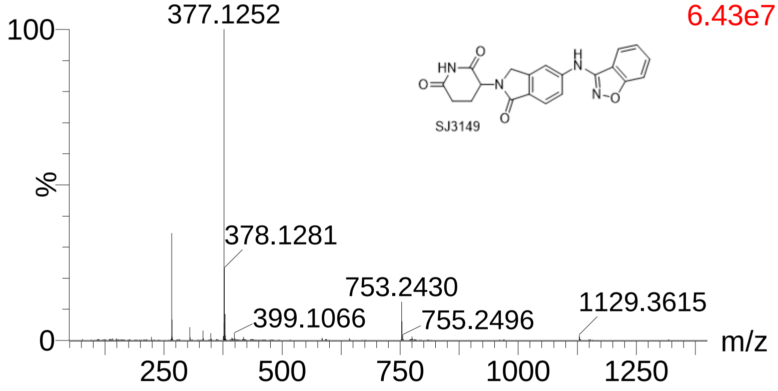

Supplementary Tables

Supplementary Table 1| Summary of data from Fig. 2. Data represents the average of at least 3 technical replicates and SD, unless otherwise stated. *Data represents the average of at least 3 biological replicates and SEM.

	SJ3149	SJ0040	SJ7095	Lenalidomide
CK1α DC_{50} μM (Hibit, 4 hrs)	0.001 ± 0.0008	0.01 ± 0.0018	0.035 ± 0.0036	>10 ± 18.4
CK1α D_{max} % (10 μM)	95.5 ± 0.13	88.3 ± 1.3	82.2 ± 1.1	42 ± 1.6
CK1α Rate (10 μM)	3.51	1.8	1.33	0.43
CK1α:CRBN NanoBret (2 hrs) EC_{50} μM, maxfold	0.1 ± 0.06, 38.0 ± 1.5	0.1 ± 0.07, 12.53 ± 0.28	0.31 ± 0.19, 6.21 ± 0.3	0.18 ± 0.1, 3.80 ± 0.4
IKZF1 DC_{50} (4 hrs) μM	>10 ± NA	>10 ± NA	0.069 ± 0.014	0.033 ± 0.007
IKZF1 D_{max} % (10 μM)	<10% ± 6.0	<10% ± 3.8	83.7 ± 5.7	64.5 ± 7.4
CRBN FP IC_{50} μM*	0.844 ± 0.034	1.217 ± 0.291	1.323 ± 0.833	1.604 ± 0.420

Supplementary Table 3| Data collection and refinement statistics for the structure of CK1α+CRBN+DDB1 in complex with SJ3149 (PDB code: 8G66)

	CK1α+CRBN+DDB1+SJ3149
**Data collection**	
Space group	*P*1
Cell dimensions	
a, *b*, *c* (Å)	88.66, 109.71, 112.34
α, β, γ (°)	105.49, 94.41, 100.03
Resolution (Å)	48.25-3.45 (3.54-3.45)*
R_{merge}	0.177 (0.770)
I / σI	8.6 (1.8)
Completeness (%)	97.1 (90.2)
Redundancy	4.5 (3.4)
**Refinement**	
Resolution (Å)	48.25-3.45
No. reflections	49,744
R_{work} / R_{free}	0.213/0.273
No. atoms	
Protein	21,267
Ligand/ion	28/2
Water	0
B-factors	
Protein	122.95
Ligand/ion	98.92/113.15
Water	NA
R.m.s. deviations	
Bond lengths (Å)	0.052
Bond angles (°)	1.01

Data was collected from two crystals. *Values in parentheses are for highest-resolution shell. Atomic coordinates and structure factors have been deposited in the Protein Data Bank with accession code 8G66.

Supplementary Table 4| In-vitro mouse and human ADME data for SJ3149.

	**SJ3149**
Aqueous solubility (µM)	36
MDCK-MDR1 AB/BA (nm/s)	277/365
Microsome stability $t_{1/2}$: mouse/human (h)	>10
Hepatocyte stability $t_{1/2}$: mouse/human (min)	>216
Plasma stability: mouse/human (h)	>10/8.5
Plasma protein binding: mouse/human (%)	96.2/97.7

Supplementary Methods

MOLM-13 Cell Viability Assay and Lenalidomide Competition Assay. Exponentially growing MOLM-13 cells were plated in Corning 8804BC white 384-well assay plates at the appropriate cell number per well and incubated overnight at 37 °C in a humidified 5 % CO_2 incubator. Compounds were transferred to the assay plate from a dose-response plate using a Labcyte Echo 650 Acoustic Liquid Handler (Beckman Coulter, USA). Cytotoxicity was determined following 72 h of incubation using Promega's CellTiter-Glo reagent according to the manufacturer's recommendation. Luminescence was measured on the Envision plate reader (PerkinElmer, USA). Raw luminescence relative light unit (RLU) values for each compound at each concentration were normalized to obtain % activity using the following equation (1): 100 × [(mean(negctrl) – compound)/(mean (negctrl) – mean (posctrl))]. The % activity was converted to % viability using the following equation (2): (100 – % activity), and then pooled from replicate experiments prior to fitting. Here, negctrl and posctrl refer to the negative (DMSO) and positive controls (staurosporine) on each plate. Dose-response curves were fit to the Hill equation using the GraphPad Prism (version 10). For the lenalidomide competition assay, the assay plates containing cells were pre-treated with lenalidomide (40 μM) and incubated for 30 minutes prior to addition of the compounds.

General Chemistry Methods and Materials. All reagents and solvents were obtained from commercially available sources and were used without further purification. 3-(4-(aminomethyl)-1-oxoisoindolin-2-yl)piperidine-2,6-dione HCl salt, Catalog#:SYNNAAX405291 was obtained from Synnovator, INC., 3-(5-amino-1-oxoisoindolin-2-yl)piperidine-2,6-dione (EN300-1272845) and 3-(5-bromo-1-oxoisoindolin-2-yl)piperidine-2,6-dione (EN300-6478894) were obtained from Enamine. Reactions were set up in air and carried out under nitrogen atmosphere. Nuclear magnetic resonance (NMR) spectra were obtained on a Bruker NMR spectrometer at 500 MHz for 1H-NMR spectra and 125 MHz for 13C-NMR spectra. Chemical shifts (ppm) are reported relative to the solvent peak. Signals are designated as follows: s, singlet; d, doublet; dd, doublet of doublet; t, triplet; q, quadruplet; m, multiplet. Coupling constants (J) are expressed in Hertz. High resolution mass spectral data were obtained on a Waters Xevo G2 QTof mass spectrometer. The purity of final compounds was performed on an Acquity UPLC BEH C18 1.7 μm, 2.1 x 50 mm column (Waters

Corporation, Milford, MA) using an Acquity ultra performance liquid chromatography system. The flow rate was 0.7 mL/min. The sample injection volume was 3 μL. The UPLC column was maintained at 50 °C and the gradient program started at 90% A (0.1% formic acid in MilliQ H2O), changed to 95% B (0.1% formic acid in Acetonitrile) over 2.5 min, held for 0.35 minutes, then to 90% A over 0.05 minutes.

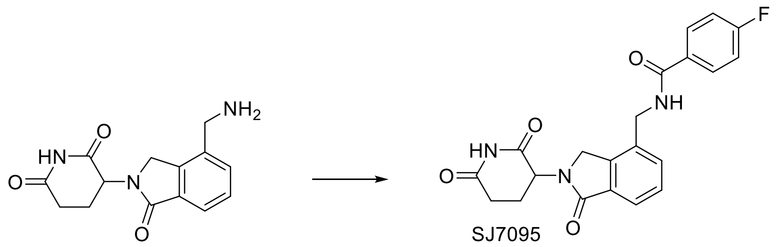

***N*-((2-(2,6-dioxopiperidin-3-yl)-1-oxoisoindolin-4-yl)methyl)-4-fluorobenzamide (SJ7095).** To a vial equipped with a stir bar was added 3-(4-(aminomethyl)-1-oxoisoindolin-2-yl)piperidine-2,6-dione HCl salt (0.092 mmol, 1.0 equiv) and THF (500 μL) followed by 4-fluorobenzoyl chloride (0.18 mmol, 2.0 equiv) and DIPEA (0.27 mmol, 3 equiv). The reaction was stirred at room temperature for 2 hr. The reaction was checked by UPLC then diluted with DMSO (1 mL). Purification was performed on the Waters purification/analytical LC/UV/ELSD system and the gradient program started at 90% A (0.1% formic acid in MilliQ H_2O), changed to 40% B (0.1% formic acid in Acetonitrile) over 10.3 min, then changed to 95% B over 2 min, held for 1 minutes, then changed to 90% A over 0.25 minutes and held for 1.75 min. The flow rate is 30 mL/min. Evaporation was carried out using a TurboVap® LV evaporator to afford *N*-((2-(2,6-dioxopiperidin-3-yl)-1-oxoisoindolin-4-yl)methyl)-4-fluorobenzamide (SJ7095, 15 mg, 0.038 mmol, 41%. Purity > 95%). ^{1}H NMR (500 MHz, DMSO) δ 10.95 (s, 1H), 9.06 (t, *J* = 5.8 Hz, 1H), 7.89 (dd, *J* = 8.4, 5.6 Hz, 2H), 7.57 (d, *J* = 7.4 Hz, 1H), 7.49 (d, *J* = 7.5 Hz, 1H), 7.44 (t, *J* = 7.5 Hz, 1H), 7.28 – 7.21 (m, 2H), 5.08 (dd, *J* = 13.3, 5.1 Hz, 1H), 4.53 – 4.44 (m, 3H), 4.37 (d, *J* = 17.3 Hz, 1H), 2.86 (ddd, *J* = 18.1, 13.4, 5.3 Hz, 1H), 2.60 – 2.52 (m, 1H), 2.37 – 2.27 (m, 1H), 2.02 – 1.92 (m, 1H). ^{13}C NMR (125 MHz, MeOD) δ 173.27, 170.77, 170.05, 167.65, 164.92 (d, *J* = 251 Hz), 140.50, 134.01,

131.56, 131.27, 130.25, 129.60 (d, J = 9.07 Hz), 128.42, 122.11, 115.09 (d, J = 22.3 Hz), 52.26, 46.78, 39.92, 30.96, 22.73. LCMS (m/z) M+H = 396.5. HRMS (m/z): calcd for $C_{21}H_{18}FN_3O_4$ + H: 396.1359; found: 396.1348 [M + H].

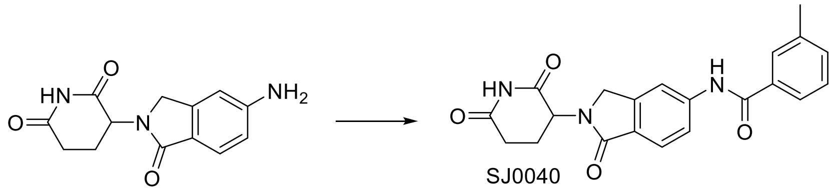

***N*-(2-(2,6-dioxopiperidin-3-yl)-1-oxoisoindolin-5-yl)-3-methylbenzamide (SJ0040).** To a vial equipped with a stir bar was added 3-(5-amino-1-oxoisoindolin-2-yl)piperidine-2,6-dione (0.075 mmol, 1.0 equiv) and THF (500 µL) followed by 3-methylbenzoyl chloride (0.150 mmol, 2.0 equiv) and DIPEA (0.225 mmol, 3 equiv). The reaction was stirred at room temperature for 2 hrs. The reaction was checked by UPLC then diluted with DMSO (1 mL). Purification was performed on the Waters purification/analytical LC/UV/ELSD system and the gradient program started at 90% A (0.1% formic acid in MilliQ H_2O), changed to 40% B (0.1% formic acid in Acetonitrile) over 10.3 min, then changed to 95% B over 2 min, held for 1 minutes, then changed to 90% A over 0.25 minutes and held for 1.75 min. The flow rate is 30 mL/min. Evaporation was carried out using a TurboVap® LV evaporator to afford

N-(2-(2,6-dioxopiperidin-3-yl)-1-oxoisoindolin-5-yl)-3-methylbenzamide (SJ0040, 14 mg, 0.038 mmol, 50%, Purity > 95%). ^{1}H NMR (500 MHz, DMSO) δ 11.02 (s, 1H), 10.55 (s, 1H), 8.17 (d, J = 1.8 Hz, 1H), 7.86 (dd, J = 8.3, 1.8 Hz, 1H), 7.83 – 7.76 (m, 2H), 7.74 (d, J = 8.3 Hz, 1H), 7.50 – 7.43 (m, 2H), 5.12 (dd, J = 13.3, 5.2 Hz, 1H), 4.50 (d, J = 17.2 Hz, 1H), 4.36 (d, J = 17.2 Hz, 1H), 2.94 (ddd, J = 17.3, 13.7, 5.4 Hz, 1H), 2.68 – 2.59 (m, 1H), 2.48 – 2.34 (m, 4H), 2.11 – 1.99 (m, 1H). ^{13}C NMR (125 MHz, DMSO) δ 173.42, 171.59, 168.37, 166.60, 143.58, 142.96, 138.30, 135.12, 132.92, 128.87, 128.68, 127.17, 125.42, 123.99, 120.41, 114.86, 52.05, 47.67, 31.69, 22.99, 21.43. LCMS (m/z) M+H = 378.5. HRMS (m/z): calcd for $C_{21}H_{19}N_3O_4$ + H: 378.1454; found: 378.1451 [M + H].

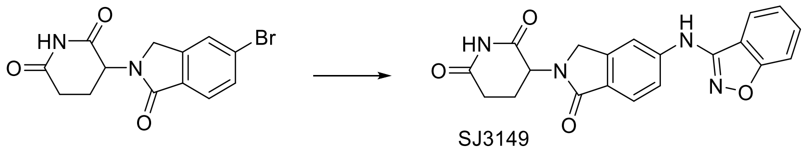

SJ3149

3-(5-(benzo[d]isoxazol-3-ylamino)-1-oxoisoindolin-2-yl)piperidine-2,6-dione (SJ3149). To a vial equipped with a stir bar was added 3-(5-bromo-1-oxoisoindolin-2-yl)piperidine-2,6-dione (0.31 mmol, 1.0 equiv), benzo[d]29soxazole-3-amine (0.46 mmol, 1.5 equiv) purchased from Combi-Blocks Inc., (9,9-dimethyl-9H-xanthene-4,5-diyl)bis(diphenylphosphane) (Xantphos, 0.031 mmol, 0.1 equiv), Tris(dibenzylideneacetone)dipalladium (0.031 mmol, 0.1 equiv) and potassium carbonate (0.62 mmol, 2.0 equiv), followed by DMSO (3 mL). The reaction was heated to 120 ^{o}C in a microwave reactor for 1 hr. The reaction mixture was checked by UPLC and filtered through a pile of celite. Purification was performed on the Waters purification/analytical LC/UV/ELSD system and the gradient program started at 80% A (0.1% formic acid in MilliQ H_2O), changed to 60% B (0.1% formic acid in Acetonitrile) over 10.5 min, then changed to 95% B over 1.5 min, held for 0.80 minutes, then changed to 80% A over 0.2 minutes and held for 2 min. The flow rate is 30 mL/min. Evaporation was carried out using a TurboVap® LV evaporator to afford 3-(5-(benzo[d]isoxazol-3-ylamino)-1-oxoisoindolin-2-yl)piperidine-2,6-dione (SJ3149, 22 mg, 0.058 mmol, 19%, Purity > 95%). ^{1}H NMR (500 MHz, DMSO) δ 10.92 (s, 1H), 9.93 (s, 1H), 8.15 – 8.07 (m, 1H), 7.95 (d, *J* = 1.8 Hz, 1H), 7.71 – 7.62 (m, 2H), 7.63 – 7.56 (m, 2H), 7.35 (ddd, *J* = 8.0, 5.7, 2.2 Hz, 1H), 5.03 (dd, *J* = 13.3, 5.2 Hz, 1H), 4.42 (d, *J* = 17.1 Hz, 1H), 4.28 (d, *J* = 17.1 Hz, 1H), 2.85 (ddd, *J* = 17.2, 13.6, 5.4 Hz, 1H), 2.58 – 2.51 (m, 1H), 2.34 – 2.24 (m, 1H), 1.98 – 1.90 (m, 1H). ^{13}C NMR (125 MHz, DMSO) δ 173.41, 171.67, 168.47, 161.62, 155.36, 144.46, 144.39, 131.25, 124.86, 124.41, 123.46, 122.18, 117.93, 116.89, 111.93, 110.16, 51.99, 47.66, 31.72, 23.06. LCMS (m/z) M+H = 377.4. HRMS (m/z): calcd for $C_{20}H_{16}N_4O_4$ + H: 377.1250; found: 377.1252 [M + H].

ADME Methods

Aqueous solubility assay: Solubility assays were conducted using a Biomek FX lab automation workstation (Beckman Coulter, Inc., Fullerton, CA) and μSOL Evolution software (pION Inc., Woburn, MA). In a 96-well microplate (Cat. No: 3363, Corning Incorporated, Salt Lake, UT), 10 μL of a 10 mM test compound stock in DMSO was added to 190 μL 1-propanol (Thermo Scientific, spectroscopy, Cat. No: 434360010, Fair Lawn, NJ) to create a reference stock plate. From this reference stock plate, 5 μL solutions were mixed with 70 μL 1-propanol and 75 μL Dulbecco's Phosphate Buffered Saline (DPBS, 1X, GibcoTM, Cat. No: 14190-144, Thermo Fisher Scientifics, Waltham, MA) to generate the reference plate (high sensitivity UV plate, Cat. No: 110286, pION Inc., Woburn, MA). In a 96-well storage plate (Cat. No: 201276-100, Agilent, Santa Clara, CA), 6 μL of a 10 mM test compound stock was added to 600 μL buffer, mixed, sealed, and incubated at room temperature for 18 hours. Following incubation, the suspension was filtered through a 96-well filter plate (PVDF, 0.2 μM, Cat. No: 110037, pION Inc., Woburn, MA). The filtrate (75 μL) was combined with 75 μL 1-propanol to create the sample plate. UV spectra of the reference and sample plates were then read, and calculations based on the area under the curve (AUC) of the UV spectra were performed using μSOL Evolution software. All compounds were tested in triplicate wells.

MDCKII-MDR1 cell-based permeability assay: High-throughput MDCKIIMDR1 permeability assay was performed in the Transwell® 0.4 μm polycarbonate membrane 96-well system with modified methods[1,2], MDCKIIMDR1 cells were maintained at 37 °C in a humidified incubator with an atmosphere of 5% CO_2. The cells were cultured in 75 cm^2 flasks with Eagle's Minimum Essential Medium (EMEM) containing 10% fetal bovine serum (FBS), 1% non-essential amino acids (NEAA), 100 units/mL of penicillin, and 100 μg/mL of streptomycin. The MDCKIIMDR1 cells were seeded onto inserts at a density of 1×10^4 cells/insert separately. The medium in the wells was changed every other day, and the trans epithelial electrical resistance (TEER) value was measured

using a REMS Autosampler (World Precision Instruments, Sarasota, FL). MDCKIIMDR1 cells were grown for 5 days to reach a consistent confluency level monitored by epithelial voltohmmeter to confirm the intactness of the confluent polarized monolayer. For transport experiments, each cultured monolayer on the 96-well plate was washed twice with a transport buffer (HBSS/25 mM HEPES, pH 7.4). The cell-based permeability assay was initiated by the addition of each compound solution (10 μmol/L) into inserts (apical side, A) or receivers (basolateral side, B). After incubated for 1 hour at 37 °C, fractions were collected from receivers (if apical to basal permeability) or inserts (if basal to apical permeability), and concentrations were assessed by UPLC/MS (Waters; Milford, MA). All compounds were tested in triplicates. The A→B (or B→A) apparent permeability coefficients (Papp) of each compound were calculated using the equation (3): $Papp=dQ/dt\times 1/AC_0$, where dQ/dt equals the flux of a drug across the monolayer, the parameters of A equals the surface area of total insert well, and C_0 is the initial concentration of substrate in the donor compartment. The efflux ratio was determined by dividing the P_{app} in the B-A direction by the P_{app} in the A-B direction. An efflux ratio greater than two suggests that a given substrate is actively transported across the cell monolayer membrane.

Liver microsomes stability assay[3,4]: NADPH regenerating agent solutions A and B (Corning Gentest™, Cat. No: 451220) were purchased from Discovery Labware (Woburn, MA). Mouse liver microsomes (CD-1, pooled, Gibco™, Cat. No: MSMCPL) were obtained from Thermo Fisher Scientific (Fredrick, MD). Pooled human liver microsomes (mixed gender, Cat. No: E0630) were purchased from XenoTech (Lenexa, KS). Stock solutions of test compounds were prepared at 10 mM in DMSO. Sample preparation for microsomal stability was modified from Di's publications[3,4]. 50 nL of stock solutions were transferred into six 96-well microplates (labelled as 0, 0.25, 0.5, 1, 2, and 4 hours) by Labcyte Echo 650 acoustic dispenser (Beckman Coulter Life Sciences, Indianapolis, IN). All compounds were tested in triplicate per time point. Concentrated human or mouse liver microsomes (20 mg/mL protein concentration) and 0.5 M EDTA (Fluka Cat. No: 03690, Sigma-

Aldrich, Saint Louis, MO) were diluted into 0.1 M potassium phosphate buffer (PBS, pH 7.4, KH_2PO_4, Cat. No: P284-500; K_2HPO_4, Cat. No: BP363-500, Fisher Scientific, Fair Lawn, NJ) and mixed well. 40 μL of this solution was transferred into the above compounds plates. For the time 0 plate, 3 fold (v:v) cold acetonitrile with warfarin (400 ng/mL) as the internal standard was added to each well, followed by addition of 10 μL NADPH regenerating agent (mixing NADPH solutions A and B in PBS, pH 7.4) and no incubation. For the other five time points' plates, 10 μL NADPH regenerating agent mixer was added to each well to initiate the reaction, the plate was sealed and incubated at 37° C on an orbital shaker (100 rpm) for the required time, followed by quenching of the reaction by adding 3-fold volume of cold acetonitrile with internal standard to each well. The final concentration of each component applied in this reaction was liver microsome protein at 0.5 mg/mL, EDTA at 1 mM, compound at 10 µM, NADPH A at 1.3 mM, and NADPH B at 0.4 U/mL. After quenching, all plates were securely sealed and thoroughly mixed at 600 rpm for 10 minutes. Subsequently, centrifugation was performed at 4000 rpm for 20 minutes. The resulting supernatants were transferred to analytical plates and diluted with a 1:1 ratio of MilliQ water for subsequent analysis using UPLC–MS/MS. The assessment of metabolic stability involved determining the half-life through a least-squares fit of multiple time points, employing first-order kinetics.

Plasma stability assay[5]: The degradation of human or mouse plasma was assessed by monitoring the gradual reduction of the parent compound over specified time intervals (0, 3, 24, and 48 hours). Human and mouse plasma samples were sourced from GeneTex (Irvine, CA). Test compound stock solutions, prepared at 10 mM in DMSO, were dispensed into triplicate wells of a 96-well microplate using the Labcyte Echo 650 dispenser. For plates, excluding the time 0-hour plate, plasma was directly added to achieve a final compound concentration of 10 μM. The plate was sealed, incubated at 37°C on an orbital shaker (100 rpm) for the required duration, and then quenched with cold acetonitrile, incorporating warfarin as the internal standard. In the case of the time 0-hour plate, cold acetonitrile with the internal standard was added to each well before introducing plasma, eliminating

the need for further incubation. Following quenching, all plates were thoroughly mixed at 600 rpm for 10 minutes and centrifuged at 4000 rpm for 20 minutes. Supernatants were transferred to analytical plates and appropriately diluted with a 1:1 ratio of MilliQ water for subsequent UPLC–MS/MS analysis. Plasma stability was evaluated by determining the half-life through a least-squares fit of multiple time points based on first-order kinetics.

Plasma protein binding assay: Test compound stock solutions were prepared at a concentration of 10 mM in DMSO. A Single-Use RED (Rapid Equilibrium Dialysis) device was acquired from Thermo Scientific (Cat. No: 99006, Rockford, IL). The sample preparation for plasma protein binding followed a modified version of Waters' method[6]. After thawing, plasma was centrifuged at 1000 rpm for 10 minutes to eliminate any particulates. Each compound was then prepared at a concentration of 10 µM in either human or mouse plasma. In the plasma protein binding assessment, 300 µL of spiked plasma solutions were introduced into the sample chamber (indicated by the red ring), while 550 µL of DPBS was added to the adjacent chamber. The plate was sealed and incubated at 37°C on an orbital shaker (100 rpm) for 4 hours. Post-incubation, 50 µL aliquots were withdrawn from each side of the insert and dispensed into a 96-well storage plate. To create analytically identical sample matrices (matrix matching), an equivalent volume of blank plasma or DPBS was added to the necessary wells, followed by quenching with 3-fold (v:v) cold acetonitrile containing warfarin as the internal standard. Subsequently, the sealed plates were well-mixed at 600 rpm for 10 minutes and then centrifuged at 4000 rpm for 20 minutes. Supernatants were transferred to analytical plates and appropriately diluted 1:1 ratio with Millipore water for UPLC–MS/MS analysis. Test compound concentrations were quantified in both the buffer and plasma chambers by determining peak areas relative to the internal standard. The percentage of the test compound bound to plasma was calculated using the following equation (4): %Free = (Concentration buffer chamber / concentration plasma chamber) x 100% and equation (5) %Bound = 100% - %Free.

UHPLC-MS/MS System: Chromatographic separation was performed on an Acquity UPLC BEH C18 1.7 µm, 2.1 x 50 mm column (Waters Corporation, Milford, MA) using a Sciex ExionLC™ with 6500+ Qtrap system. Data were acquired using Analyst v 1.7 and analyzed using the OS software suite. The UPLC column was maintained at 55 °C. Mobile phase A was 0.1% formic acid in MilliQ H2O and Mobile phase B was 0.1% formic acid in acetonitrile. The flow rate was 0.9 mL/min with a gradient of 0-0.2 min, B% 1-1%; 0.2-0.5min, B% 1-50%; 0.5-1.6 min, B% 50-95%; 1.6-1.95 min, B% 95-95%; 1.95-1.96 min, B% 95-1%; and 1.96-2.2 min, B% 1-1%. The mass spectrometer was operated in positive-ion mode with electrospray ionization. MRM transition m/z 376.9>266.0 was applied for the quantification of SJ3149 in the assays of liver microsome stability, plasma stability and plasma protein binding. The following MS parameters were employed for analyte measurements: ion spray voltage 5 kV, temperature 550 °C, gas 1 and gas 2 were 60, entrance potential (EP): 10 V, declustering potential (DP): 50 V, collision energy (CE): 27 V, and collision cell exit potential (CXP): 16 V.

Metabolic Stability in mouse and human cryopreserved hepatocytes. 10 mM test compounds were provided by Compound Sample Group. Diluted 10 mM test compounds and 30 mM positive control compounds (7-ethoxycoumarin) to 1 mM and 3 mM with DMSO in 96-well plates. Diluted 1 mM test compounds and 3 mM positive control compounds to 100 µM and 300 µM dosing solutions with ACN. Cryopreserved cells were thawed, isolated and suspended in Incubation Medium, then diluted with pre-warmed Incubation Medium to 0.5 × 10^6cells/mL. Added 198 µL of pre-warmed cell suspensions in 96-well plates. Transferred 125 µL of stop solution (acetonitrile containing 200 ng/mL tolbutamide and 200 ng/mL labetalol as internal standards) in a set of pre-labeled 96-well plates. Spiked 2 µL dosing solution to each well of 96-well plates in duplicates. For T0 Samples, mixed to achieve a homogenous suspension for about 1 min, then immediately transferred 25 µL of each sample into well containing 125 µL of ice-cold stop solution followed by mixing. Incubated all plates at 37°C in a 95% humidified incubator at 5% CO_2 to start the reactions

with constant shaking at about 600 rpm. At 15, 30, 60 and 90 min, mixed samples and then transferred 25 µL of each sample at each time point to well containing 125 µL of ice-cold stop solution followed by mixing. Medium Control (MC) sample plates (labeled as T0-MC and T90-MC) were prepared at T0 and T90 by adding the same components to each well except cell suspensions. At each corresponding time point, stopped the reactions by removing the plates from incubator and mixing with 125 µL of ice-cold stop solution. Vortexed the plates immediately on a plate shaker at 500 rpm for 10 minutes. Then, centrifuged all sample plates at 3220 x g for 20 min at 4°C. After centrifugation, 80 µL/well of supernatant in the sample plates were transferred to another set of pre-labeled 96-well plates which containing 240 µL of ultra pure water according to the plate map. Analytical plates were sealed and stored at 4°C until LC-MS/MS analysis. **Thawing Medium:** Williams' Medium E containing 5% fetal bovine serum and 30% Percoll solution and other supplements. **Incubation Medium:** Williams' Medium E (no phenol red) containing 2 mM L-Glutamine and 25 mM HEPES. **Stop Solution:** Acetonitrile containing 200 ng/mL tolbutamide and labetalol as internal standards. **Dilution Solution:** Ultra-pure water. The remaining percents of test articles after incubation were calculated by the follow equations:

Equation (6):

$$\%\ \text{Remaining (at Appointed Time)} = \frac{\text{Peak Area Ratios of Test Article versus Internal Standard at Appointed Time}}{\text{Peak Area Ratios of Test Article versus Internal Standard at 0min}} \times 100\ \%$$

Use equation of first order kinetics to calculate $t_{1/2}$ and Clint:

Equation of first order kinetics:

Equation (7):

$$C_t = C_0 \cdot e^{-k \cdot t}$$

$$C_t = \frac{1}{2} C_0,\ t_{1/2} = \frac{\ln 2}{k} = \frac{0.693}{k}.$$

When

Equation (8):

$CL_{int\ (hep)}$ = k / million cells per mL

Equation (9):

$CL_{int\ (liver)} = CL_{int\ (hep)}$* liver weight (g/kg body weight) *hepatocellularity

Proteomics Data Analysis

Identification/quantification of proteins

The MS/MS raw data were processed by a tag-based hybrid search engine, JUMP[7]. The data was searched against the UniProt human database (104,374 protein entries; downloaded in April 2020) concatenated with a reversed decoy database for evaluating False Discovery Rate (FDR). Searches were performed using a 15 ppm mass tolerance for precursor ions, fully tryptic restriction with two maximal missed cleavages, three maximal modification sites, and the assignment of b, and y ions. TMT tags on lysine residues and N-termini (+304.2071453 Da for TMT16 and TMT18) were used for static modifications. In addition, oxidation of Methionine (Met) was considered as a dynamic modification (+15.99492 Da). Putative peptide spectral matches (PSMs) were filtered by mass accuracy and then grouped by precursor ion charge state and further filtered by JUMP-based matching scores, Jscore and ΔJn, to achieve the protein FDR below 1%. For quantification, TMT intensities of reporter ions were extracted, filtered, normalized, and summarized into peptide and protein levels.

Differential expression analysis of proteins

Identification of differentially expressed proteins were conducted by the limma R package[8]. To control multiple testing, the Benjamini-Hochberg method was employed. Proteins with an adjusted

p value of < 0.05 and $\log_2$ fold change of > 2-fold of standard deviation (SD) were defined as differentially expressed.

REFERENCES

1. Larson, B.; Banks, P.; Sherman, H.; Rothenberg, M. Automation of Cell-Based Drug Absorption Assays in 96-Well Format Using Permeable Support Systems. *J Lab Autom*, *17* (3), 222–23 (2012).
2. Uchida, M.; Fukazawa, T.; Yamazaki, Y.; Hashimoto, H.; Miyamoto, Y. A Modified Fast (4 Day) 96-Well Plate Caco-2 Permeability Assay. *Journal of Pharmacological and Toxicological Methods*, *59* (1), 39–43 (2009).
3. Di, L.; Kerns, E. H.; Li, S. Q.; Petusky, S. L. High Throughput Microsomal Stability Assay for Insoluble Compounds. *International Journal of Pharmaceutics 317* (1), 54–60 (2006).
4. Di, L.; Kerns, E.; Ma, X.; Huang, Y.; Carter, G. Applications of High Throughput Microsomal Stability Assay in Drug Discovery. *CCHTS*, *11* (6), 469–476 (2008).
5. Di, L., et al., Development and application of high throughput plasma stability assay for drug discovery. *Int J Pharm*. 297(1-2), 110-9 (2005).
6. Waters, NJ, et al. "Validation of a rapid equilibrium dialysis approach for the measurement of plasma protein binding." *J Pharm Sci*. 97.10, 4586-95 (2008).
7. Wang, X., Li, Y., Wu, Z., Wang, H., Tan, H., and Peng, J. JUMP: a tag-based database search tool for peptide identification with high sensitivity and accuracy. *Molecular & Cellular Proteomics* 13:3663-3673 (2014).
8. Ritchie, M. E., Phipson, B., Wu, D., Hu, Y., Law, C.W., Shi, W., Smyth, G.K. limma powers differential expression analyses for RNA-sequencing and microarray studies. *Nucleic acids research*. 43(7):e47 (2015).

Uncropped blots for Supplementary Figures

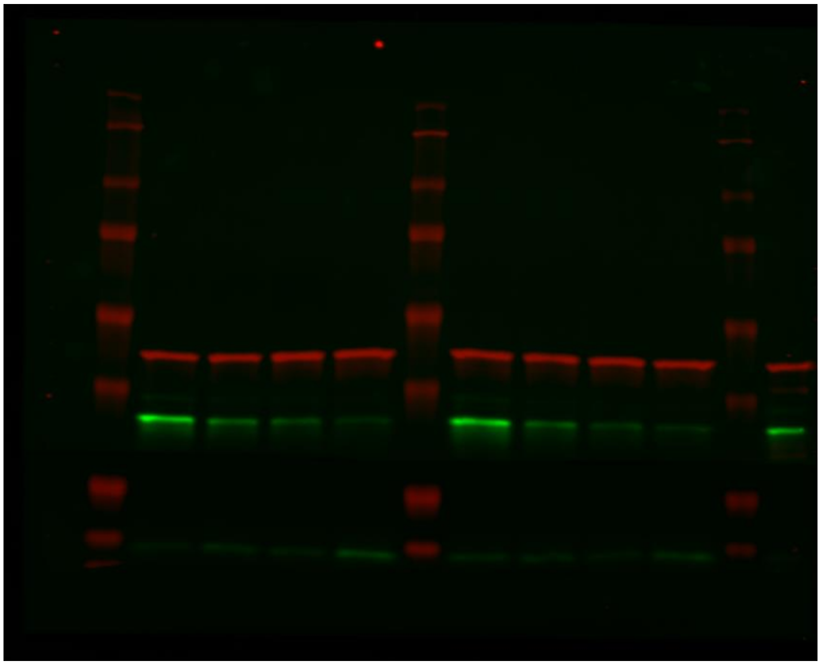

Supplementary Fig. 3d: 2 replicates: probed for actin, CK1a (35 kDa), p21 (p21 data not included in figure)

lane	sample
1	NTC A
2	CK1a #1 A
3	CK1a #2 A
4	CK1a #3 A
5	NTC B
6	CK1a #1 B
7	CK1a #2 B
8	CK1a #3 B

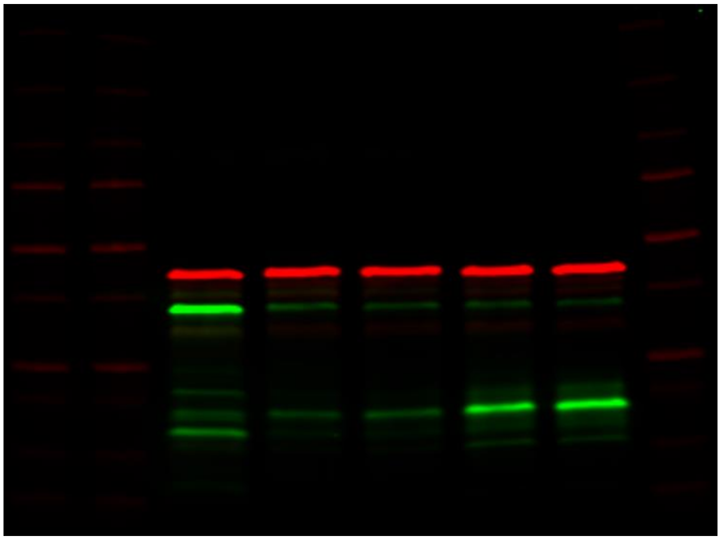

250
150
100
75
50
37
25
20
15

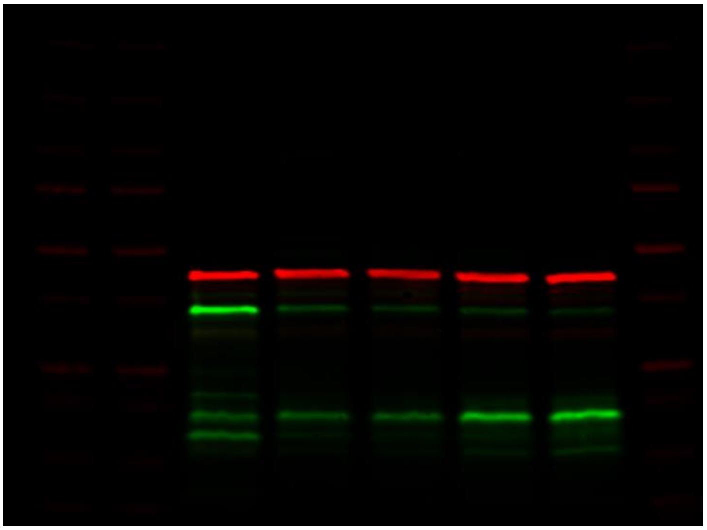

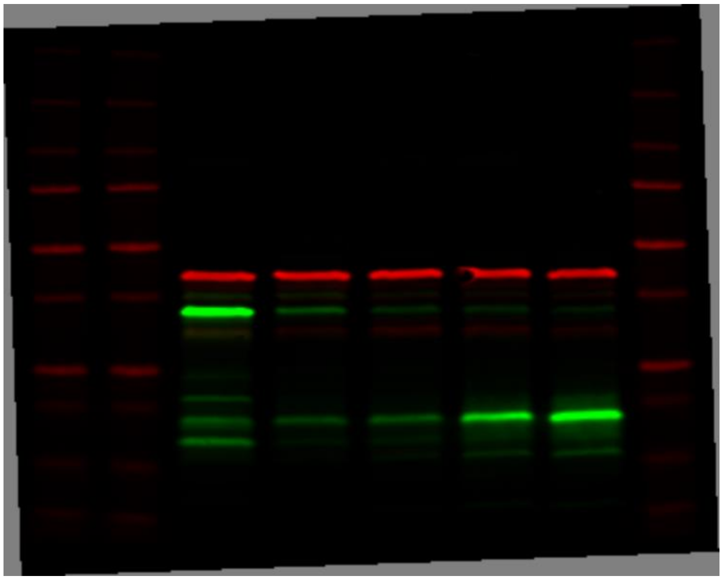

Supplementary Fig. 6c: 3 western blots probed for actin (45), CK1a (35), p21 (21)

lane	sample
1	no treatment
2	1 hr
3	2 hrs
4	4 hrs
5	8 hrs

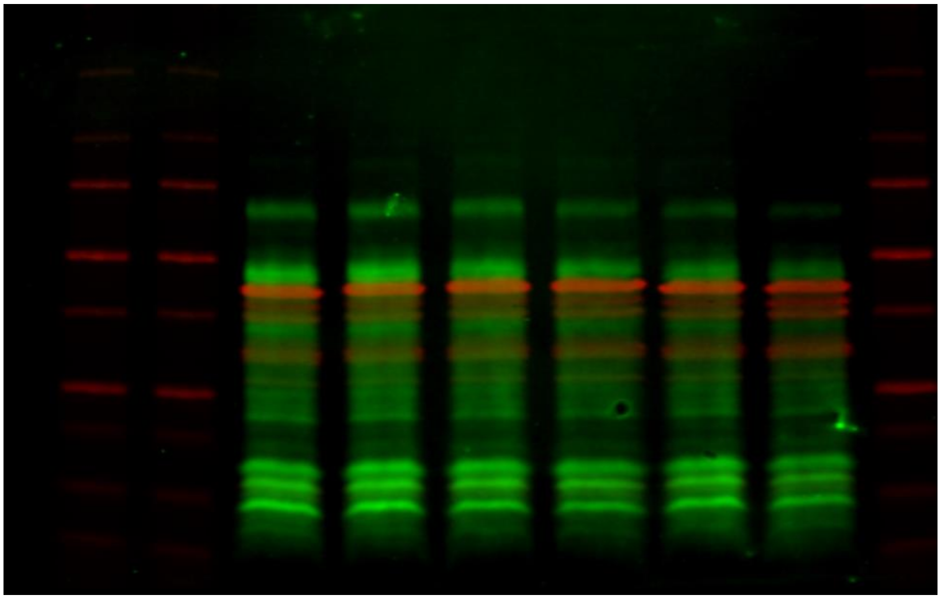

250kDa
150
100
75
50
37
25
20
15
10

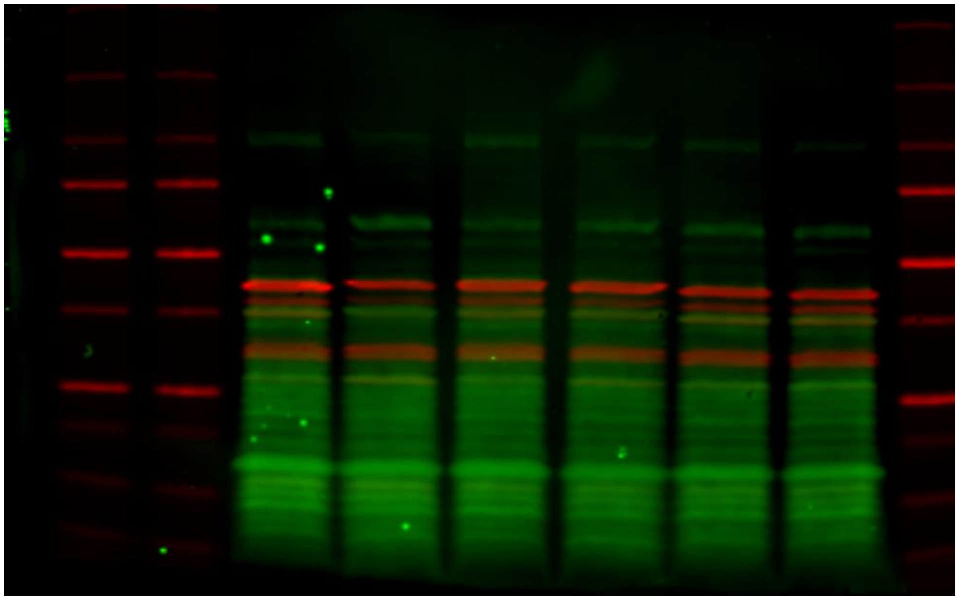

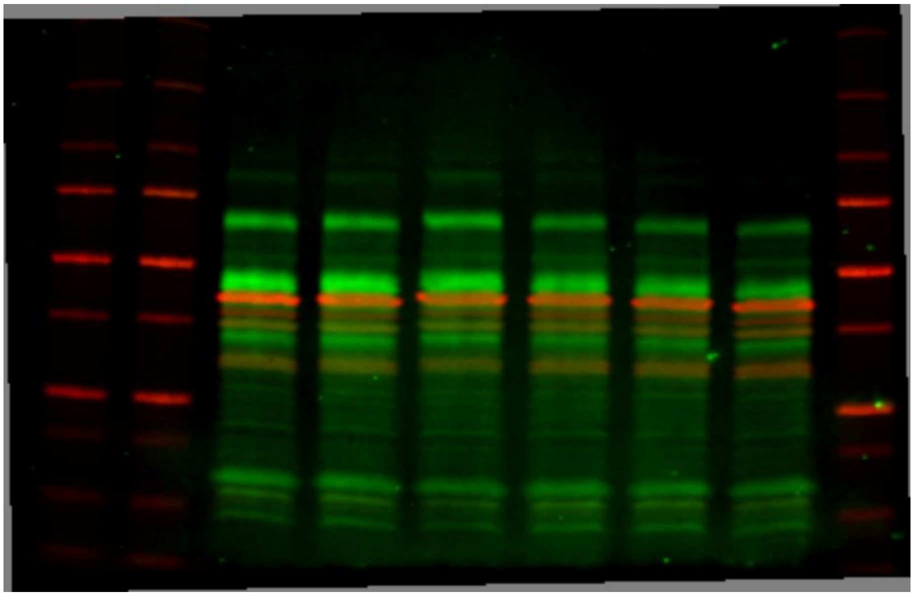

Supplementary Fig. 6d: 3 western blots probed for IKZF2 (70), actin (45)

lane	sample
1	DMSO
2	1 nM
3	10 nM
4	100 nM
5	1 uM
6	10 uM

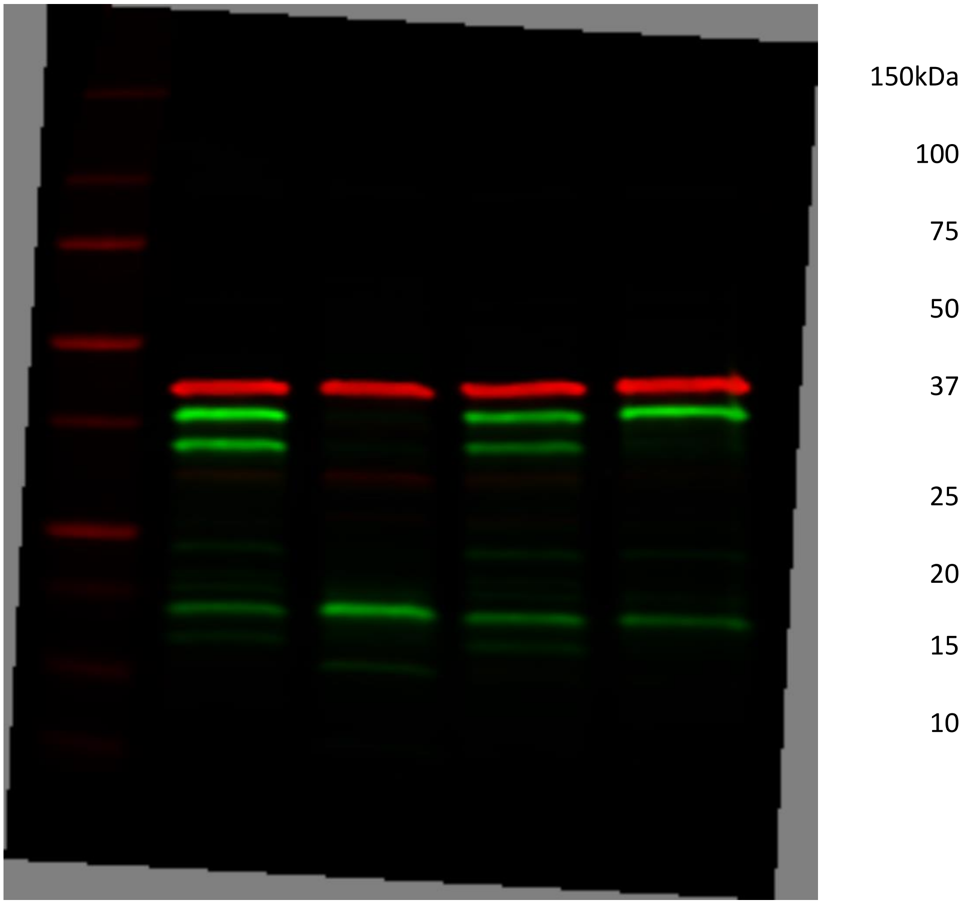

Supplementary Fig. 7a: probed for actin (45), CK1a (FLAG: 40, WT: 35), p21 (21)

lane	sample
1	FLAG-CK1a WT DMSO
2	FLAG-CK1a WT 1 uM SJ3149
3	FLAG-CK1a G40N DMSO
4	FLAG-CK1a G40N 1 uM SJ3149
