# Supplementary material for: Selective CK1α degraders exert antiproliferative activity against a broad range of human cancer cell lines
Source: Nat Commun. 2024 Jan 16;15:482. doi: 10.1038/s41467-024-44698-1 (PMC10791743; doi:10.1038/s41467-024-44698-1)
Supplement: Supplementary file 2 — Reporting Summary [file 41467_2024_44698_MOESM2_ESM.pdf]

nature portfolio

Corresponding author(s): *Double-anonymous peer review submissions: write DAPR and your manuscript number here instead of author names.*

Last updated by author(s): *YYYY-MM-DD*

Reporting Summary

Nature Portfolio wishes to improve the reproducibility of the work that we publish. This form provides structure for consistency and transparency in reporting. For further information on Nature Portfolio policies, see our Editorial Policies and the Editorial Policy Checklist.

Statistics

For all statistical analyses, confirm that the following items are present in the figure legend, table legend, main text, or Methods section.

n/a	Confirmed	
☐	☒	The exact sample size (*n*) for each experimental group/condition, given as a discrete number and unit of measurement
☐	☒	A statement on whether measurements were taken from distinct samples or whether the same sample was measured repeatedly
☐	☒	The statistical test(s) used AND whether they are one- or two-sided<br>*Only common tests should be described solely by name; describe more complex techniques in the Methods section.*
☒	☐	A description of all covariates tested
☒	☐	A description of any assumptions or corrections, such as tests of normality and adjustment for multiple comparisons
☐	☒	A full description of the statistical parameters including central tendency (e.g. means) or other basic estimates (e.g. regression coefficient) AND variation (e.g. standard deviation) or associated estimates of uncertainty (e.g. confidence intervals)
☐	☒	For null hypothesis testing, the test statistic (e.g. *F*, *t*, *r*) with confidence intervals, effect sizes, degrees of freedom and *P* value noted<br>*Give P values as exact values whenever suitable.*
☒	☐	For Bayesian analysis, information on the choice of priors and Markov chain Monte Carlo settings
☒	☐	For hierarchical and complex designs, identification of the appropriate level for tests and full reporting of outcomes
☐	☒	Estimates of effect sizes (e.g. Cohen's *d*, Pearson's *r*), indicating how they were calculated

Our web collection on statistics for biologists contains articles on many of the points above.

Software and code

Policy information about availability of computer code

Data collection	The following softwares were used for data collection, processing and refinement for the crystallography experiments: Sergui, XDS, XSCALE, COOT and Phenix. Schrodinger Maestro was used for molecular modeling. BD Diva was used for flow cytometry. Image Studio version 5.2 used to image western blots
Data analysis	GraphPad Prism 10 was used for data plotting and statistical analysis.<br>FlowJo version 10.9 was used for flow cytometry analysis.<br>IDBS XLfit5 (IDBS, Guildford, UK) was used for data analysis in Figure 3.<br>Phoenix WinNonlin® (Version 8.3) was used for PK analysis.<br>Image Studio version 5.2 used to analyze western blots

For manuscripts utilizing custom algorithms or software that are central to the research but not yet described in published literature, software must be made available to editors and reviewers. We strongly encourage code deposition in a community repository (e.g. GitHub). See the Nature Portfolio guidelines for submitting code & software for further information.

Data

Policy information about availability of data

All manuscripts must include a data availability statement. This statement should provide the following information, where applicable:

- Accession codes, unique identifiers, or web links for publicly available datasets
- A description of any restrictions on data availability
- For clinical datasets or third party data, please ensure that the statement adheres to our policy

Proteomics datasets discussed in the manuscript are available from the ProteomeXchange Consortium via the PRIDE partner repository. The datasets have been assigned accession numbers of PXD045170, PXD045228 and PXD045230, corresponding to the experiments related to SJ7095, SJ0040, and SJ3149, respectively. The atomic coordinates and structure factors have been deposited into the Protein Data Bank with accession codes 8G66 (Quaternary complex of CK1α+CRBNΔ1-40 +DDB1ΔBPB+SJ3149). DepMap database used to analyze cells' dependency on CK1a. All other data supporting the findings of this study are available within the article and its supplementary files. Any additional requests for information can be directed to the corresponding authors. Source data are provided with this paper.

Research involving human participants, their data, or biological material

Policy information about studies with human participants or human data. See also policy information about sex, gender (identity/presentation), and sexual orientation and race, ethnicity and racism.

Reporting on sex and gender	There were no human research participants in this study
Reporting on race, ethnicity, or other socially relevant groupings	There were no human research participants in this study
Population characteristics	There were no human research participants in this study
Recruitment	There were no human research participants in this study
Ethics oversight	There were no human research participants in this study

Note that full information on the approval of the study protocol must also be provided in the manuscript.

Field-specific reporting

Please select the one below that is the best fit for your research. If you are not sure, read the appropriate sections before making your selection.

[x] Life sciences   [ ] Behavioural & social sciences   [ ] Ecological, evolutionary & environmental sciences

For a reference copy of the document with all sections, see nature.com/documents/nr-reporting-summary-flat.pdf

Life sciences study design

All studies must disclose on these points even when the disclosure is negative.

Sample size	No sample size calculations were performed. Most cell line based experiments (treating cells for western blot or flow cytometry) were n=3, as that is a generally accepted sample size for technical replicates and statistical analysis. This logic was also used for the sample size of 3 in the PD experiment, and to avoid being wasteful with animals for initial studies.
Data exclusions	One treatment group (n=3) was excluded from the PD experiment after results were analyzed. We decided that the 100 mg/kg dosing group results were too inconsistent between animals and complicated the data presentation unnecessarily, and removed it from the final figures. One additional control group sample was removed from the data presentation due to inefficient sample collection, and one control animal died during the engraftment process before treatments began.
Replication	Several biochemical experiments were replicated 2-3 times. Occasionally 1 replicate experiment would not match the results from previous attempts but this occurred mainly due to human error or low abundance of a protein. In those cases that result was excluded because there were enough successful replicates that it was not needed. In vivo experiments were not replicated.
Randomization	Cell culture experiments were not randomized as all samples from the same replicate experiment used cells from the same culture. NSG mice were not randomized to ensure that the comparison groups are balanced for other variables.
Blinding	It would not be appropriate to blind samples in the experiments performed. In the PD study for example, mice had to be treated multiple times with the same dose for 2 days. Blinding would have made it overly complicated by involving more people and steps unnecessarily, and was not beneficial to the experiment.

Reporting for specific materials, systems and methods

We require information from authors about some types of materials, experimental systems and methods used in many studies. Here, indicate whether each material, system or method listed is relevant to your study. If you are not sure if a list item applies to your research, read the appropriate section before selecting a response.

Materials & experimental systems

n/a	Involved in the study
☐	☒ Antibodies
☐	☒ Eukaryotic cell lines
☒	☐ Palaeontology and archaeology
☐	☒ Animals and other organisms
☒	☐ Clinical data
☒	☐ Dual use research of concern
☒	☐ Plants

Methods

n/a	Involved in the study
☒	☐ ChIP-seq
☐	☒ Flow cytometry
☒	☐ MRI-based neuroimaging

Antibodies

Antibodies used

1. rabbit anti-CK1α, ab206652, Abcam
2. rabbit anti-p21, 2947S, Cell Signaling Technology
3. rabbit anti-Helios, 42427, Cell Signaling Technology
4. mouse anti-beta actin, 3700S, Cell Signaling Technology
5. goat anti-rabbit IRDye 800CW, 926-32211, LI-COR
6. goat anti-mouse IRDye 680RD, 926-68070, LI-COR
7. APC Annexin V, 640920, BioLegend

Validation

1. knockout validated by manufacturer. from website: HEK-293T Casein Kinase 1 alpha KO whole cell lysate compared to unedited cell lysate. band detected at 36 kDa in unedited cells but not in KO lysate. "The lysates were kindly provided by our collaborator Dr. Yi Rao, Peking University."
2. knockout validated by manufacturer. from website: "Western blot analysis from control HeLa cells (lane 1) or p21 Waf1/Cip1 knockout HeLa cells (lane 2) using p21 Waf1/Cip1 (12D1) Rabbit mAb (upper) or β-Actin (D6A8) Rabbit mAb #8457 (lower). The absence of signal in the p21 Waf1/Cip1 knockout HeLa cells confirms specificity of the antibody for p21 Waf1/Cip1."
3. overexpression transfection validated by manufacturer. "Western blot analysis of extracts from 293T cells, untransfected (-) or transfected with a construct expressing Myc-tagged full-length human Helios (hHelios-Myc; +), using Helios (D8W4X) XP® Rabbit mAb."
4. recombinant isoform expression validated by manufacturer. "Western blot analysis of recombinant Actin isoforms using β-Actin (8H10D10) Mouse mAb (upper) and Pan-Actin Antibody #4968 (lower)."
7. validated for flow cytometry by manufacturer. "Human T leukemia cell line Jurkat, treated (left) or non-treated (right) with BioLegend's anti-human CD95 (EOS9.1) mAb (Cat. No. 305704) for 4 hours, then stained with Annexin V- APC and Helix NP Green (Cat. No. 425303 at 5 nM) in Annexin V Binding buffer for 15 minutes at 25°C."

Eukaryotic cell lines

Policy information about cell lines and Sex and Gender in Research

Cell line source(s)

MOLM-13 cells were obtained from DSMZ (ACC 554)
HNT-34 cells were purchased from DSMZ (ACC 600)
Kasumi-3 cells were purchased from ATCC (CRL-2725)
UCSD-AML1 cells were purchased from DSMZ (ACC 691)
TF-1 cells were purchased from DSMZ (ACC 334)
MHH-CALL4, NALM-16, MHH-CALL-2 cells were obtained from German Collection of Microorganisms and Cell Cultures GmbH (DSMZ, Germany)
AML193 cells were purchased from DSMZ (ACC 549)
OCI-AML3 cells were purchased from DSMZ (ACC 690)
HEL cells were obtained from Dr. Charles Mullighan
PER-117 cells were obtained from Telethon Kids Institute (Perth, Australia).
HD-MB03 cell line was obtained from Drs. Milde, Witt and Deubzer
MB002 and MB004 cells were obtained from Dr Yoon-Jae Cho
5637 ATCC HTB-9
769-P ATCC CRL-1933
786-O ATCC CRL-1932
A-172 ATCC CRL-1620
A-204 ATCC HTB-82
A375 ATCC CRL-1619
A388 ATCC CRL-7905
A-427 ATCC HTB-53
A-498 ATCC HTB-44
A-549 ATCC CCL-185

A-704 ATCC HTB-45
ACHN ATCC CRL-1611
AN3 CA ATCC HTB-111
AP-1060 DSMZ ACC-593
AsPC-1 ATCC CRL-1682
AU-565 ATCC CRL-2351
BT-20 ATCC HTB-19
BT-549 ATCC HTB-122
BxPC-3 ATCC CRL-1687
C-33 A ATCC HTB-31
CAL 27 ATCC CRL-2095
CCF-STTG1 ATCC CRL-1718
CCRF-CEM ATCC CCL-119
CCRF-HSB-2 DSMZ ACC-435
COLO 205 ATCC CCL-222
COLO 829 ATCC CRL-1974
CTV-1 DSMZ ACC-40
Daoy ATCC HTB-186
DB ATCC CRL-2289
DLD-1 ATCC CCL-221
DoTc2 4510 ATCC CRL-7920
DU 145 ATCC HTB-81
DU4475 ATCC HTB-123
ES-2 ATCC CRL-1978
FaDu ATCC HTB-43
G-361 ATCC CRL-1424
HCT 116 ATCC CCL-247
HCT-15 ATCC CCL-225
HL-60 ATCC CCL-240
Hs 578T ATCC HTB-126
Hs 746T ATCC HTB-135
Hs 766T ATCC HTB-134
HT ATCC CRL-2260
HT-1080 ATCC CCL-121
HuTu 80 ATCC HTB-40
J82 ATCC HTB-1
JAR ATCC HTB-144
JM-1 ATCC CRL-10423
Jurkat E6.1 ATCC TIB-152
K-562 ATCC CCL-243
Kasumi-1 ATCC CRL-2724
KATO III ATCC HTB-103
KG-1 ATCC CCL-246
KLE ATCC CRL-1622
KU812 ATCC CRL-2099
LNCaP FGC ATCC CRL-1740
LoVo ATCC CCL-229
LS 174T ATCC CL-188
LS411N ATCC CRL-2159
MCF7 ATCC HTB-22
MeWo ATCC HTB-65
MG-63 ATCC CRL-1427
MIA PaCa-2 ATCC CRL-1420
MOLM-13 DSMZ ACC-554
MOLT-4 ATCC CRL-1582
NB4 DSMZ ACC-207
NCCIT ATCC CRL-2073
NCI-H460 ATCC HTB-177
NCI-H661 ATCC HTB-183
NCI-H82 ATCC HTB-175
NOMO-1 DSMZ ACC-542
OVCAR-3 ATCC HTB-161
PA-1 ATCC CRL-1572
PC-3 ATCC CRL-1435
PF-382 DSMZ ACC-38
PFSK-1 ATCC CRL-2060
PL-21 DSMZ ACC-536
RD ATCC CCL-136

	RKO ATCC CRL-2577<br>RL ATCC CRL-2261<br>RL95-2 ATCC CRL-1671<br>RPMI-7951 ATCC HTB-66<br>RPMI-8402 DSMZ ACC-290<br>RS4-11 ATCC CRL-1873<br>RT4 ATCC HTB-2<br>SEM DSMZ ACC-546<br>SET-2 DSMZ ACC-608<br>SHP-77 ATCC CRL-2195<br>SJCRH30 ATCC CRL-2061<br>SK-N-AS ATCC CRL-2137<br>SK-N-FI ATCC CRL-2142<br>SNU-5 ATCC CRL-5973<br>SNU-C2B ATCC CCL-250<br>SR ATCC CRL-2262<br>SU-DHL-1 ATCC CRL-2955<br>SU-DHL-6 ATCC CRL-2959<br>SUP-T1 DSMZ ACC-140<br>SW48 ATCC CCL-231<br>SW480 ATCC CCL-228<br>SW620 ATCC CCL-227<br>SW626 ATCC HTB-78<br>SW837 ATCC CCL-235<br>SW872 ATCC HTB-92<br>SW900 ATCC HTB-59<br>SW948 ATCC CCL-237<br>SW982 ATCC HTB-93<br>T24 ATCC HTB-4<br>T98G ATCC CRL-1690<br>TCCSUP ATCC HTB-5<br>THP-1 ATCC TIB-202<br>TT ATCC CRL-1803<br>U-118 MG ATCC HTB-15<br>U-2 OS ATCC HTB-96<br>U-87 MG ATCC HTB-14<br>VA-ES-BJ ATCC CRL-2138
Authentication	Cell identity was confirmed by STR profiling using PowerPlex® Fusion System (Promega).
Mycoplasma contamination	Cells were not tested
Commonly misidentified lines<br>(See ICLAC register)	no commonly misidentified lines were used

Animals and other research organisms

Policy information about studies involving animals; ARRIVE guidelines recommended for reporting animal research, and Sex and Gender in Research

Laboratory animals	Healthy Female CD1 mice (8-10 weeks old) were used for the PK experiments<br>NSG mice 6-8 weeks old were used for the PD experiment
Wild animals	No wild animals were used in this study
Reporting on sex	Not applicable. All NSG mice were female. Sex was not considered beyond female mice being most commonly used for cell line derived xenografts.
Field-collected samples	No field-collected samples were used in this study
Ethics oversight	Ethics approval by the St Jude Institutional Animal Care and Use Committee (PD study) and the Institutional Animal Ethics Committee (IAEC) for the PK Study at SAILIFE.

Note that full information on the approval of the study protocol must also be provided in the manuscript.

Flow Cytometry

Plots

Confirm that:

- [x] The axis labels state the marker and fluorochrome used (e.g. CD4-FITC).
- [x] The axis scales are clearly visible. Include numbers along axes only for bottom left plot of group (a 'group' is an analysis of identical markers).
- [x] All plots are contour plots with outliers or pseudocolor plots.
- [x] A numerical value for number of cells or percentage (with statistics) is provided.

Methodology

Sample preparation	MOLM-13 cells were treated as stated in the manuscript and incubated for 24 or 72 hours with compound. All samples were processed at the same time. After washing with Annexin-V FACS buffer, APC conjugated primary antibody was added to the samples. DAPI was added to cells after a final wash before sample analysis. Samples were kept on ice.
Instrument	BD Biosciences Fortessa 4 laser/17 color
Software	Diva software used to collect data, FlowJo used to analyze data
Cell population abundance	Cell population fractioning and sorting was based on comparison to control samples, including unstained and single-color samples and vehicle-treated control samples.
Gating strategy	FSC-A and SSC-A parameters used to gate cells and remove cell debris. Within that gate, single cells were gated along the diagonal from the origin point. In single cells, quadrants were plotted to divide APC positive above 10^3 and DAPI positive above 10^3.

- [x] Tick this box to confirm that a figure exemplifying the gating strategy is provided in the Supplementary Information.
