# Supplementary material for: Selective CK1α degraders exert antiproliferative activity against a broad range of human cancer cell lines
Source: Nat Commun. 2024 Jan 16;15:482. doi: 10.1038/s41467-024-44698-1 (PMC10791743; doi:10.1038/s41467-024-44698-1)
Supplement: Supplementary file 3 — Description of Additional Supplementary Files [file 41467_2024_44698_MOESM3_ESM.pdf]

Title: Supplementary Data 1
Description:

a. Collected IC_{50} values and cell line mutation status
b. Cell Lines Information

Title: Supplementary Movie 1.
Description: Morph of CK1α domain rotation when bound to SJ3149 (8G66) vs. lenalidomide (5FQD).
